# 4D Pointwise Terrestrial Laser Scanning Calibration: Radiometric Calibration of Point Clouds

**DOI:** 10.3390/s25227035

**Published:** 2025-11-18

**Authors:** Mansoor Sabzali, Lloyd Pilgrim

**Affiliations:** Surveying Discipline, School of Engineering, University of Newcastle, Newcastle, NSW 2308, Australia; lloyd.pilgrim@newcastle.edu.au

**Keywords:** accuracy, color, incidence angle, LiDAR range equation, neural network method, precision, range, 4D pointwise TLS radiometric calibration

## Abstract

**Highlights:**

**What are the main findings?**

- A novel framework for the pointwise radiometric calibration of terrestrial laser scanning (TLS) is presented, which is a combination of the LiDAR range equation, texture-dependent LiDAR cross-section determination, and a neural network technique.
- This method significantly enhances the radiometric resolution of TLS on color targets, with accuracy improvements of 31–49% across different color patches and precision improvements of approximately 97% within the same color patch for four TLS devices.

**What are the implications of the main findings?**

- TLS intensity attributes can be identified as a standardized fourth dimension in addition to the 3D spatial point clouds for more reliable reflectivity-based analysis.
- This framework demonstrates the potential path towards more robust 4D TLS calibration, where standard radiometric values from various target geometries (target materials, roughness, albedo, and edgy and tilted surfaces) are strictly required.

**Abstract:**

Terrestrial Laser Scanners (TLS), as monostatic LiDAR systems, emit and receive laser pulses through a single aperture, which ensures the simultaneous measurement of signal geometry and intensity. The relative intensity of a signal, defined as the ratio of received to transmitted power, directly describes the strength and quality of the reflected signal and the corresponding radiometric uncertainty of individual points. The LiDAR range equation provides the physical connection for characterizing signal strength as a function of reflectivity and other spatial parameters. In this research, theoretical developments of the texture-dependent LiDAR range equation, in conjunction with a neural network method, are presented. The two-step approach aims to improve the accuracy of signal intensities by enhancing signal reflectivity estimation and the precision of signal intensities by reducing their sensitivity to variations in spatial characteristics—range and incidence angle. This establishes the intensity as the standard fourth dimension of the 3D point cloud based on the inherent target quality. For validation, four terrestrial laser scanners—Leica ScanStation P50, Leica ScanStation C10, Leica RTC360, and Trimble X9—are evaluated. Results demonstrate significant improvements of at least 40% in accuracy and 97% in precision for the color intensities of individual points across the devices. This research enables a 4D TLS point cloud calibration framework for further investigations on other internal and external geometries of targets (target materials, roughness, albedo, and edgy and tilted surfaces), which allows the standardization of radiometric values.

## 1. Introduction

### 1.1. Problem Background

With the advent of the first generation of LiDAR (Light Detection and Ranging), data acquisition approaches in photogrammetry and engineering geodesy have been entirely revolutionized. LiDAR has different classifications, but in terms of aperture types, it is categorized as monostatic or bistatic LiDAR, having the same or separate apertures for emitting and receiving light, respectively [1,2]. A terrestrial laser scanner (TLS) is classified as a monostatic LiDAR due to its single-aperture design.

TLS is fundamentally an active sensor that uses visible or infrared waves of the electromagnetic spectrum (EM) to capture spatial and radiometric data through the availability of the reflected signal from a scene (i.e., each point in a point cloud provides both 3D spatial coordinates and radiometric information). One of the systematic error sources degrading the point clouds’ quality—spatially and radiometrically—is the unknown impact of the target geometry and surface properties [3]. The evaluation of the radiometric uncertainty of TLS point clouds—signal strength or intensity of the returned pulse from sample scanned targets—is the focus of this research (with the main emphasis on color targets).

It was reported that points lying on identical color patches (with similar target quality) do not uniformly scatter reflectance [4,5]. To comprehend the radiometric influences received from individual points, it is crucial to investigate the amount of energy received via the energy link equation by experimenting with variant sample targets under multiple scanning arrangements. This energy link budget equation is satisfied through the implementation of the LiDAR range equation. This method simultaneously takes into account the reflectivity and geometric characteristics—range and incidence angle—of a single point. Here, various samples of color targets (Macbeth color chart) under two scanning conditions—orthogonal and inclined—are assessed for accuracy using four scanner devices.

### 1.2. Significance and Purposes

The current work aims to address signal intensity as a pointwise factor that varies with respect to range, incidence angle, and reflectivity. Conventional methods of intensity modeling limit the consideration of the spatial characteristics of a single point and neglect the influence of surface reflection. Since points sharing the same target quality do not scatter the signal uniformly, a detailed study of the individual points on the target is important.

To reinforce this, for the first time, the LiDAR range equation is introduced as a well-established framework for assessing the point-to-point radiometric uncertainty in terrestrial laser scanning. The innovative pointwise radiometric calibration of TLS is achieved through an appropriate determination of the LiDAR cross-section, accounting for the surface reflection of the target properties, and by developing neural network techniques to incorporate weightings of the spatial dimensions of a single point—range, and incidence angle. The two-step approach expressed here deals with both challenges together to enhance the accuracy and precision of the intensity values for a single pulse. The proposed framework ultimately aims to standardize the intensity values within each color patch.

For data collection, a laboratory calibration setup was established under controlled environmental conditions using four terrestrial laser scanners—Leica ScanStation P50, Leica ScanStation C10, Leica RTC360 (Leica Geosystems, Heerbrugg, Switzerland), and Trimble X9 (Trimble, Westminster, CO, USA)—under orthogonal and inclined scanning conditions. Due to the varying wavelengths and multiple beam characteristics, the selected devices provide a noteworthy validation under different scanning environments. Note that the calibration conditions were also isolated in terms of reflectivity from any unexpected interfering light sources during scanning. It is worth clarifying that the effect of refractivity along the laser line is insignificant within the short range of scanning [6]. A significant aspect of the data experiments is that scanning was conducted under maximum default resolution and in “Scan Only” mode (with no attached RGB camera data). The study reveals that the RGB values derived from single laser pulses cannot replicate true color equivalents, dissimilar to those derived from cameras. They are simply alternative representations of signal intensity (intensity-based color reproduction values).

The investigation of the results indicates the success of the proposed two-step approach in determining the color-dependent LiDAR cross-section and data-driven neural network techniques across different color intensities. The accuracy experiences improvements between 31% and 47%, while precision shows improvements of 97% or higher. Accuracy refers to the accurate reflectivity modeling of the surface corresponding to the inherent color targets, whereas precision encompasses the pointwise intensity modeling by addressing the range and incidence angle of the returned pulse. The methodologies presented here provide a radiometric calibration framework that is independent of scanning and target geometry—range and incidence angle, but dependent on reflectivity. The findings underscore that the 3D point coordinates of TLS are complemented by the standard 1D intensity coordinate with respect to the signal strength. This part aims to provide a future pathway toward fully standardized 4D point clouds calibration that accounts for additional target characteristics (material, roughness, and edgy or tilted geometries).

## 2. Related Works

**Figure 1:**
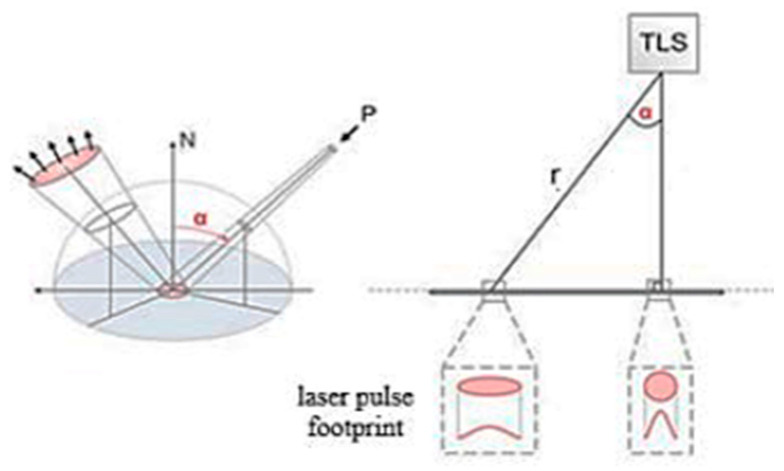
Incidence angle α and range r attached to the single measured point P [11].

The uncertainty of TLS deliverables is highly dependent on the reflected signal’s strength. Several factors affect the signal strength, including the reflectivity of the surface, range, and incidence angle of a single-point observation [7,8,9,10]. Not only do these factors affect the geometric uncertainty of 3D point coordinates, but they also create a deviation in radiometric uncertainty. To clarify these contributing parameters, the range r is the physical value and can be measured (Figure 1). However, the incidence angle α is a non-physical value and can be described theoretically as the angle between the transmitted laser beam vector L and the surface-normal vector $\vec{N\;}$.

The ideal condition of the incidence angle is zero when the shape of the laser spot footprint is circular rather than elliptical. Then, this corresponds to a zero reflected signal angle. This occasionally occurs for a single point measurement, only when the orthogonal condition of scanning is satisfied. Studies [11,12] expressed that an incidence angle larger than 60° significantly affects the overall 3D point cloud precision, and the presence of noise increases by 20% as the incidence angle rises. Nevertheless, at smaller incidence angles, range becomes the dominant factor influencing signal strength [7,10]. Therefore, the behavior of the reflected signal, primarily influenced by range and subsequently by the incidence angle, plays a critical role in determining the strength of the backscattered signal.

The investigation of both effects—range and incidence angle—on the intensity was initially evaluated by using the laser radar range equation [13]. In this experiment, six different laboratory setups at varying ranges and incidence angles were proposed to test the reflected intensities against the known reflectivity of the targets. These findings confirm that the range has a greater impact on intensity values than the incidence angle. Accordingly, the intensity function was logarithmically approximated with respect to range, following the inverse-square law, with the impact of the incidence angle considered negligible.

Specifically, color targets have been additionally examined in previous studies for processing geometric range displacements through the availability of the signal strength. The experiment considers variant illumination conditions [12]. In the experimental setups, a Macbeth color checker chart was scanned, and the results were compared to the reference plane. The Macbeth color checker chart is a unique test pattern scientifically designed to help determine the true color balance of any color rendition system (https://hollynorth.com/product/macbeth-chart/) (accessed on 15 September 2025). In conclusion, it was noted that the ambient light behind any color leads to meaningful errors in the range measurements since it influences the noise level of the reflected signal. Finally, the corresponding geometric corrections in the measured range for different colors were determined. Moreover, the research classified the colors into two groups as follows: lighter colors that indicate high reflectance, better point density, and minimal noise with smaller geometric range distortions; and darker colors that show low reflectance, poorer point density, and higher noise with larger geometric range distortions [14]. J. Clark et al. [15] also proposed three varying range setups—close (less than 4 m), near (4 m to 6 m), and far range (7 m to 8 m)—to assess the impacts of color variations on range measurements. It was highlighted that ideal conditions occur at arbitrary range observations when geometric compensation is internally applied within the scanners. Additional studies reported the effects of color targets on range distortions under various experimental setups [16,17,18].

Former studies have also highlighted the comparison of point intensities with radiometric imagery data, considering both range and incidence angle. D. Wujanz [19] retrieved a mathematical relationship between three variables—range, incidence angle, and reflectivity—and intensities. In the computational principle, intensity through a polynomial function was formulated and calculated by interpreting the projection matrix of the scanner as the image and using the intensity values derived from the 2D image. The function eventually allowed for transforming the measured intensities into pseudo-quotient values of reflection. A reasonable reduction in the standard deviation of intensity values was achieved after the correction of the range, incidence angle, and reflectivity.

Building on former findings, recent research on the colorization of 3D point clouds aimed to utilize 2D images extracted from scanned data. The experiment evaluated various image quality methods with respect to color accuracy, sharpness, information capacity, and noise [20]. The study noted that basic challenges in color reproduction (e.g., measured differences in color, white balance, and exposure) could be mitigated during the data processing stages; however, the detailed aspects of image production (e.g., sharpness and noise) were less controllable, reflecting the inherent limitations in scanner construction [20]. A comparative analysis between TLS intensity data and RGB camera data in [21]—based on identifying the coordinates of target centers—indicated that the existing systematic errors are sometimes greater than the beam divergence of the scanner.

Balaguer-Puig et al. [22] established the connection between TLS intensity and color properties on the Macbeth color chart. Through this comparison, a reasonable relationship between surface reflectivity and color intensity values can be observed. According to these outcomes, the current study proposes a generic framework for intensity standardization based on color reflectivity, formulated through a color-dependent LiDAR range equation. This framework is further supported by a data-driven neural network approach that accounts for variations in range and incidence angle. The two-step technique introduces a pointwise correction procedure for 4D radiometric calibration in TLS. This methodology not only investigates color properties but also systematically enables its extension to other target geometries. Ultimately, the study offers a unique contribution to radiometric TLS error modeling in order to guarantee consistent intensity values corresponding to the reflected backscattered signal.

## 3. TLS Background

### 3.1. TLS Deliverables

TLS is a terrestrial laser-based instrument that delivers 3D point coordinates in 3D spherical coordinates. In principle, TLS is a very high-speed and movable total station which can capture millions of points in a second as a result of measuring three spherical coordinates—range r, horizontal angle h, and vertical angle v—from the returned signals reflected from individual points [23]. The mathematical conversion is applied from 3D spherical coordinates to 3D Cartesian coordinates as follows:

(1) $$\left[\begin{array}{c} x\\ y\\ z \end{array}\right]=\left[\begin{array}{c} r\cos v\cos h\\ r\cos v\sin h\\ r\sin v \end{array}\right],$$

**Figure 2:**
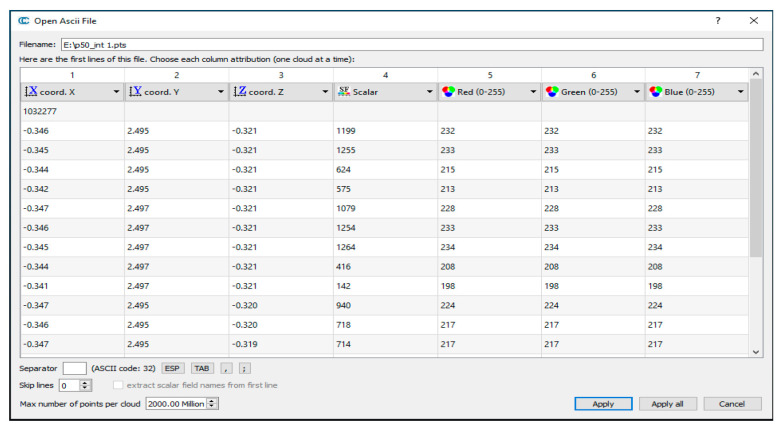
The pts format of TLS deliverables (dataset: Leica ScanStation C10, and color: yellow). A randomly selected point cloud is shown, for demonstration purposes only acquired from CloudCompare v2.7.0 (https://www.cloudcompare.org; accessed on 15 September 2025).

Apart from the 3D spatial coordinates, every measured point includes the following four additional radiometric values (Figure 2): a scalar field and the red, green, and blue (RGB) color components.

The scalar field (i.e., intensity or grayscale values) is a numerical attribute assigned to each point, describing the strength of the reflected signal. These values depend on numerous factors such as surface roughness, material properties, incidence angle, etc. In contrast, the red, green, and blue color components (RGB) are always expressed on a scale between 0 and 255, where 0 indicates no reflected intensity, but 255 represents the maximum reflected intensity. To relate these perceptual color estimates to physical measurements, a conversion from RGB values to intensity values is required (Appendix A). However, the radiometric products of terrestrial laser scanning are generally uncalibrated deliverables and cannot be directly applied to reflectivity-related tasks. Accordingly, the uncertainty assessment of these non-spatial values within TLS calibration studies is referred to as radiometric calibration. The current study focuses on the fourth dimension of TLS observations—intensity value—alongside the conventional 3D spatial coordinates.

One of the major systematic error sources impacting those radiometric values is the external geometry of the scanned targets (edged and tilted surfaces) and/or their inherent characteristics (e.g., roughness, material, color, albedo, etc.)—commonly referred to as object and surface-related issues. Although other geometries concerning the instrument and scanning configuration must be simultaneously involved in comprehensive TLS calibration studies, the key focus of the current research is on one of the inherent features of the object-related issues—the color of targets—regardless of the exterior and interior topographies of the targets.

### 3.2. Object- and Surface-Related Issues

A comprehensive calibration study for terrestrial laser scanning embraces the incorporation of the following four geometries: instrument, laser, scanning, and target geometries. One of the important geometries among the four relates to the characteristics and properties of the scanned targets. When the laser strikes the surface of the targeted object, it experiences several optical phenomena. Reflectivity is one of the dominating factors leading to the varying power of reflected signals. Several elements play integral roles in the varying reflectance behavior of sample targets, namely, material [24], roughness [25,26,27,28], color (the discussion point in the current work), albedo [29], and the geometric configuration of the surface, such as the edged and tilted properties [30]. The major emphasis, however, is on the laser striking the target surface, with reduced influence from other optical interferences such as atmospheric effects along the propagation path [6].

**Figure 3:**
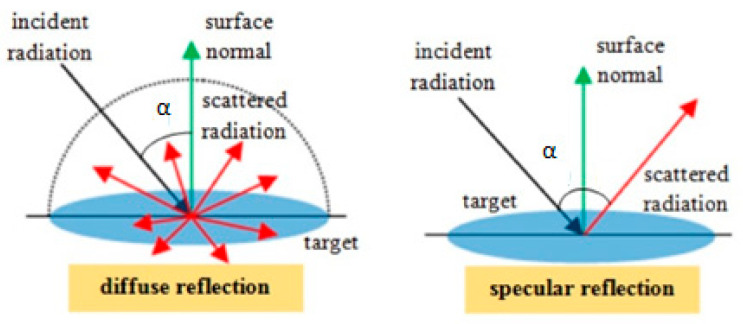
Different patterns of surface reflectivity (i.e., diffuse or specular reflection [32]).

Reflectivity in optics is a measure with regard to the ability of a surface to reflect the radiation (i.e., here, the radiation is the laser illumination, typically within the visible or infrared domain of the electromagnetic spectrum (EM)). Hence, reflectiveness is defined as the quality or capability of the surface being reflective. Generally, reflectivity occurs in two general patterns [31,32] (Figure 3).

When the beam generates specular reflectivity (mirror or regular reflectivity), meaning that the returned signal is uniformly illuminated like the emitted signal, the reflection is equal and specular in the same direction. Specular returns are frequently referred to as a glint in research activities, but this type of reflection is unlikely to happen in nature. The alternative pattern of the reflected signal is scattered over a large volume and non-uniformly in different directions. This pattern is called diffuse reflection. The (perfect) diffuse reflection is referred to as Lambertian reflectance. Although the perfect diffuse is unlikely to take place in nature, Lambertian reflectance is assumed in most reflectance-related research for LiDAR research investigations [33,34]. Thus, to better quantify the radiometric quantities of terrestrial laser scanning, the geometry of the laser striking the target surfaces alongside the geometry of the targets are two simultaneous issues. Therefore, a laboratory test arrangement must be conducted to address variant sample targets.

In summary, the points with the same target characteristics (e.g., color) can exhibit varying reflectivity, meaning that neighboring points on the same surface do not necessarily scatter the laser signal identically and uniformly. Variations in range, incidence angle, reflectivity, and other degradation parameters influence the strength of individual point returns, and they lead to different radiometric qualities. These factors directly affect the received power and the corresponding signal-to-noise ratio (SNR), resulting in inconsistent intensity values across the identical color patch (Figure 2). To address these challenges, this study proposes an amended methodology that integrates pointwise intensity modeling—based on the LiDAR range equation—in conjunction with a neural network, resulting in a rigorous 4D radiometric TLS calibration. This framework can further account for various factors influencing individual point intensity measurements, including the other object- or surface-related issues.

## 4. Methods

A pointwise study of reflected power is proposed for intensity modeling in terrestrial laser scanning (TLS). This approach enhances the accuracy and precision of the fourth dimension of point clouds, namely, the intensity values associated with each point observation. To address this challenge, both radiometric variation (reflectivity) and geometric variation (range and incidence angle) must be considered simultaneously, since even through points share the same target quality (e.g., color), they can produce inconsistent reflected laser power. A two-step methodology, based on a modified theoretical development on the LiDAR range equation, is presented to standardize intensity values with respect to color targets. In the first step, the color-dependent LiDAR cross-section is accurately determined to distinguish between intensities according to their corresponding reflectivity. In the second step, neural network algorithms are incorporated to minimize the sensitivity of the derived pointwise intensity to spatial resolution effects.

The LiDAR range equation is an energy link budget interpretation which specifies the attenuation of the signal due to its propagation and the other possible deteriorating factors (i.e., it relates the received to the transmitted power of the signal, considering additional factors that might degrade signal strength). The comprehensive LiDAR range equation is written as follows [1,33,34]:

(2) $$P_{R}=P_{T}\frac{\Omega }{A_{illum}}\frac{A_{rec}}{\pi r^{2}}\eta_{atm}^{2}\eta_{sys},$$

where the received power of the laser $P_{R}$ is reduced in magnitude compared to transmitted power $P_{T\;}$ in Watts. The relationship is established through the following two ratios: the first, the area of cross-section Ω that is divided by the illumination area at the target location $A_{illum}$; and the second, the area of receiver aperture $A_{rec}$ that is divided by the effective average area illuminated by the reflection from the target $\pi r^{2}$ depending on measured range r (all areas are computed in $m^{2}$). There are two efficiency terms, as follows: $\eta_{atm}$ is the transmission efficiency through the atmosphere (atmospheric loss), and $\eta_{sys}$ is the optical efficiency of the receiver system (system loss) (both are dimensionless values). The efficiency of a system or a process is typically expressed as the ratio of useful output to total input. Notably, under ideal efficiency, the two efficiency terms are equal to one, and the equation is simplified.

The area of the receiver $A_{rec}$ ($m^{2})$ is determined as the function of the diameter of receiver $D_{rec}\;\left(m\right).$ In case of a circular aperture of a single receiver, it is generally given as follows:

(3) $$A_{rec}=\pi {\left(\frac{D_{rec}}{2}\right)}^{2},$$

The diameter of the receiver is dependent on the beam divergence ϑ (rad) and wavelength λ (m).

(4) $$D_{rec}\approx \frac{\lambda }{\vartheta },$$

Equation (4) can be expressed in different formats depending on the beam profile. Several beam profiles were defined in the literature. The most common one encountered in laser-based sensors is the Gaussian beam profile with multiple definitions of beamwidth ω. Although there is no fixed definition of beamwidth, full width at half maximum (FWHM) is one of the customary conventions used for laser scanner devices. It represents the full beamwidth where the intensity is half the maximum. For example, for the Gaussian FWHM beam profile, Equation (4) can be rewritten as $D_{rec}=1.22\frac{\lambda }{\vartheta }$ [34].

The second area of a two-way energy budget is $A_{illum}$ ($m^{2})$, the illuminated area of the actual ray beam. This area is not smaller than the diffraction limit. The diffraction limit is theoretically the smallest size at which optical systems can resolve the targets. It is assumed that the transmitted beam uniformly illuminates a circular output aperture [34]. The area is based on the diameter of the receiver $D_{rec}\;(m)$ and can be computed as follows [31,34]:

(5) $$A_{illum}\approx \pi {\left(\frac{\lambda \;r}{2\;D_{rec}}\right)}^{2},$$

**Figure 4:**
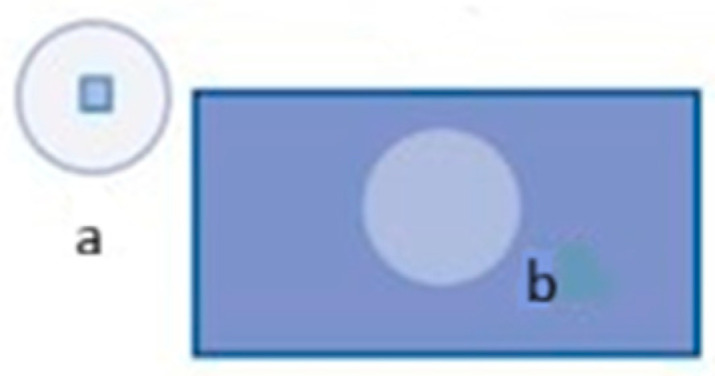
(**a**) Point targets, and (**b**) extended targets [33].

The foremost critical parameter is to obtain the LiDAR cross-sectional area for any given target. The computation of this non-physical area at the target location plays an essential role in reflected power and intensity value determination, as it is theoretically defined as a perfectly reflecting spherical area, dependent on the illumination area $A_{illum}\;(m^{2})$ and surface reflectance γ (dimensionless). The more closely the object being measured resembles a circle, the stronger the relationship between the LiDAR cross-section and the physical dimensions of the object. Accordingly, the ideal condition occurs whose illumination area is identical to the dimension of the targets $\left(\Omega =A_{illum}\right)$. This phenomenon does not occur in nature. Therefore, two underlying assumptions are substantiated. The first assumption is when the area of the targets is smaller than illumination area $\left(\Omega <A_{illum}\right)$ (i.e., smaller beamwidth)—point targets (Figure 4, condition a (the central focus of pointwise radiometric calibration)), and the second is extended targets whose area is larger than the illumination area $\left(\Omega >A_{illum}\right)$ (i.e., larger beamwidth) (Figure 4, condition b).

The other involved parameter in the LiDAR cross-section is the surface reflection. To determine the reflectivity, the dominating backscattering signal is diffuse reflectance $\gamma_{d}$ rather than specular reflectance $\gamma_{s}$ ${(\gamma }_{s}\ll \gamma_{d})$, as previously discussed in Figure 3. Then, the bidirectional reflectance distribution function (BRDF) plots of real surfaces typically display the combination of those two components equal or lower than one $\gamma =\gamma_{s}+\gamma_{d}\leq 1$. Those plots are explained via the Rayleigh condition, where a diffuse term (as the random case) decreases by an error-free term (specular) $e^{-\Lambda^{2}}$.

(6) $$\gamma_{d}={1-e}^{-\Lambda^{2}},$$

where $\Lambda =4\frac{2\pi \left(RMS\right)}{\lambda }$ is an empirical factor, whose root mean square (RMS) is a measure of surface roughness. For specular reflection, the RMS is significantly smaller than the laser wavelength, or generally negligible, whereas for Lambertian (diffuse) reflection, the RMS is in the order of the laser wavelength or larger.

When the RMS of surface roughness is similar across the target (RMS≈1), the reflectance term for all points lying on the same target properties (here color) is effectively equal to one, making the reflectivity nearly identical for all different colors. It is difficult to distinguish the surface roughness solely based on target reflectivity in terrestrial laser scanning calibration. To further account for the issue, diffuse reflectance term $\gamma_{d}$ must be scaled by the intrinsic reflectance coefficient with respect to the specific target texture k. Coefficient k is dimensionless and can be amended with respect to the proportion of incident energy reflected by a perfect Lambertian surface (i.e., this is reflected from the given color target under the propagating laser wavelength $k_{colour})$.

(7) $${\gamma_{d}}^{*}={k_{colour}(1-e}^{-\Lambda^{2}}),$$

The coefficient can be either experimentally determined or, alternatively, a standard intensity value for the given target texture at a specific wavelength can be substituted. Then, point-to-point reflectivity variations ${\gamma_{d}}^{*}\;$(dimensionless) are influenced by $k_{colour}$ and surface roughness (i.e., the intrinsic texture- (color-)dependent reflectivity). This methodology is designed to distinguish reflectivity differences based on the intrinsic surface reflectance properties of points that exhibit only slight variations in geometric parameters (Section 6.2). Therefore,

(8) $${\Omega_{d}}^{*}=4{\gamma_{d}}^{*}\pi \omega^{2}\cos\alpha ,$$

Given all the parameters of the LiDAR range equation, relative intensity I (dimensionless) is computed as follows:

(9) $$I=\frac{P_{R}}{P_{T}}=\frac{{\Omega_{d}}^{*}}{A_{illum}}\frac{A_{rec}}{\pi r^{2}},$$

According to Equation (9), the variations in geometric factors at the single-point level, namely, range and incidence angle, also contribute to fluctuations in signal strength, which are addressed in the second step of the presented method. To minimize the sensitivity of the intensity to these spatial factors, the LiDAR range equation is reformulated in terms of the weighted combination of range and incidence angle as follows:

(10) $$I=w_{1}f_{1}\left(r\right)+w_{2}f_{2}\left(\alpha \right),$$

Given Equations (8) and (9),

(11) $$f_{1}\left(r\right)=\frac{1}{r^{2}},\;\mathrm{and}\;f_{2}\left(\alpha \right)=\cos\alpha ,$$

Here, $w_{1}$ and $w_{2}$ are the corresponding weightings on the individual measurements of the range and incidence angle which are able to estimate the relative contribution of each geometric parameter to the measured intensity. These weightings are variant with respect to target and scanner geometry. A data-driven neural network algorithm is introduced to predict this complex relationship between intensity and geometric factors in order to mitigate the sensitivity of pointwise intensity to spatial variations. Therefore, intensity values I can be reparametrized as follows (Section 6.3):

(12) $$I={\gamma_{d}}^{*}\left[w_{1}\frac{1}{r^{2}}+w_{2}\cos\alpha \right],$$

Neural networks are machine learning models inspired by the structure of the human brain. They consist of multiple layers of interconnected neurons, where each neuron applies a nonlinear transformation to its inputs [35]. During training, the algorithm iteratively adjusts its internal weights to minimize the residuals between predicted and measured intensities. In principle, the proposed data-driven neural network operates on color-dependent reflectivity, range, and incidence angle as inputs, learning to estimate intensity values relative to the corresponding surface reflectivity as the output. The following four steps summarize the procedure of the pointwise radiometric calibration of TLS using the neural network approach:

1. The parameters, such as range, incidence angle, and color-dependent reflectivity obtained from the LiDAR range equation, are integrated into a feature matrix, and the output variable is determined as the intensity values.
2. The dataset is randomly divided into training (80%) and testing (20%) subsets to enable the independent evaluation of neural network performance (i.e., weightings on spatial parameters for a single point observation).
3. A feed-forward neural network with two hidden layers (each containing 10 neurons) is trained using the Levenberg–Marquardt (*trainlm*) optimization algorithm. The hidden layers use the hyperbolic tangent sigmoid (*tansig*) activation function, while the output layer employs a linear (*purelin*) function suitable for regression. As an example, training is performed with a learning rate of 0.001, a maximum of 1000 epochs, and an early stopping criterion based on validation error. Finally, the objective function minimizes the mean squared error between predicted intensity and color-dependent intensity using the LiDAR range equation (i.e., those were formerly validated through the intrinsic reflectance coefficient).
4. During the validation process, the point reflectivity (i.e., intensity) from a presumed color patch is compared against the reference reflectivity (i.e., intensity derived from neutral colors) within each dataset. This comparison provides a quantitative assessment of the improvements achieved by both the data-driven method and the physical, laser-based approach (Section 7).

For the quantitative assessment of the accuracy improvements, accuracy was estimated from the variability of repeated observations, expressed by the sample standard deviation (Bessel-corrected) [36].

(13) $$\sigma =\sqrt{\frac{1}{n-1}\sum_{i=1}^{n}({I_{i}-\bar{I})}^{2}}$$

where $\bar{I}$ is the average intensity value computed using $\bar{I}=\frac{1}{n}\sum_{i=1}^{n}I_{i}$, $I_{i}$ represents the intensity of the i-th point, and n is the total number of points within each color patch.

The accuracy assessment involves evaluating the intensity values before and after applying the color-dependent LiDAR range equation, in comparison with the corresponding standard intensity of each color. Precision evaluation is performed by analyzing the repeatability of the measured intensity values with respect to a neutral reference color patch selected for each dataset (i.e., the neutral grey patch of the Macbeth chart in each dataset exhibits the minimal deviation from the standard intensity). Accordingly, reductions in standard deviation are compared for the purpose of quantifying the improvement in measurement.

This entire formulation establishes the connection between the radiometric-based LiDAR range equation and spatial-based neural network, which allows that both geometric and radiometric effects of a single point are incorporated for rigorous 4D radiometric standardization. This two-way approach offers the calibrated intensity values that are independent of the scanning geometry and target orientation but more dependent on intrinsic target reflectance (e.g., color). This results in the consistent (standard) fourth radiometric dimension of 3D point clouds.

## 5. Data Experiment

### 5.1. Laser Study

**Figure 5:**
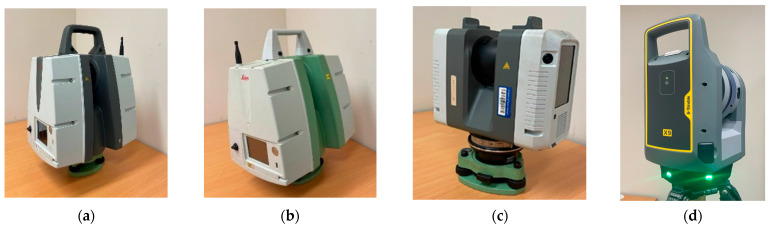
(**a**) Leica ScanStation P50, (**b**) Leica ScanStation C10, (**c**) Leica RTC360, and (**d**) Trimble X9.

Terrestrial laser scanners are active sensors that utilize the laser to capture data. Overlooking the relevant knowledge of laser geometry leads to unreliable calibration results. In the laboratory experiment proposed for this research, four scanners—Leica ScanStation P50, Leica ScanStation C10, Leica RTC360 (Leica Geosystems, Heerbrugg, Switzerland), and Trimble X9 (Trimble, Westminster, CO, USA)—are used (Figure 5). The objective is to examine the behavior of multiple wavelengths and different physical beam characteristics when interacting with the reflected signal strength from various sample targets.

Table 1 provides a summary of the technical radiometric specifications reported by the manufacturers of the employed scanners (with further explanations provided below for clarification).

**Figure 6:**
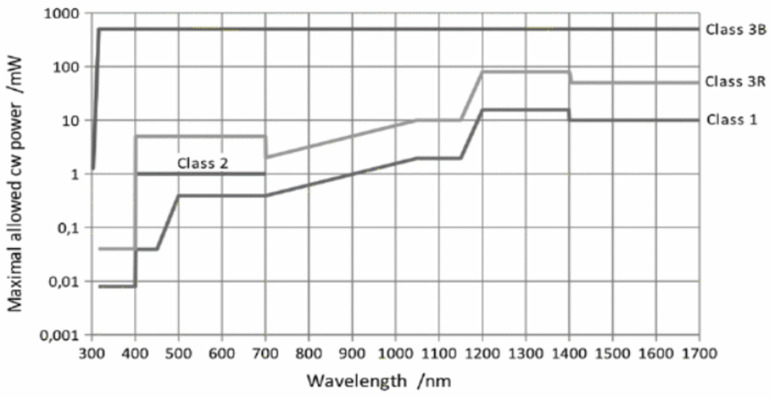
Different classifications of the laser and corresponding maximum output power (mW) [37].

Figure 6 depicts the relationship between the maximum allowed power of a continuous wave in milliwatts (mW) and different classifications of the laser with respect to the corresponding wavelengths (nm). For example, Leica ScanStation P50, with laser Class 1 and a long wavelength, potentially emits a signal with higher power than Leica ScanStation C10, with laser Class 3R and a short wavelength. Further classifications of the laser are provided here (https://en.wikipedia.org/wiki/Laser_safety) (accessed on 15 September 2025).

In addition, wavelength is a contributing factor in signal power. Among all scanners, Leica ScanStation C10 is equipped with a visible green laser that falls within the visible light spectrum (532 nm). This wavelength is suitable for bathymetric LiDAR installation since it can penetrate water and capture the sea floor. Leica ScanStation P50, Leica RTC 360, and Trimble X9 emit infrared waves (1550 nm), whose behavior is considerably diverse, and they typically interact weakly with color variations (i.e., they are not sensitive enough for color distinction, especially for visible colors); however, they are suitable for the distinction of material properties and rough surfaces and ideal for long-range scanning. As outlined above, differences in wavelengths and beam characteristics produce varying radiometric responses. This, therefore, enables a more rigorous basis for validating the proposed 4D TLS calibration practices.

### 5.2. Data Collection Steps

**Figure 7:**
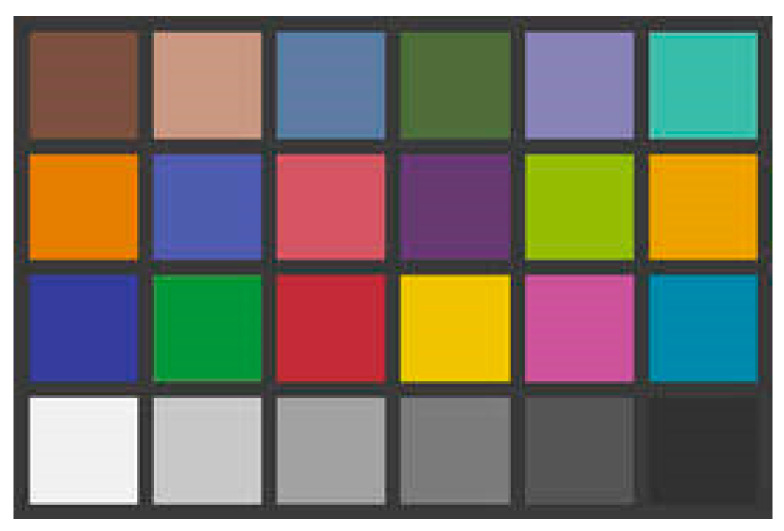
The Macbeth color chart [38].

In the following experiment, a series of sample (color) targets are tested, regardless of their inherent qualities and external formation. For data acquisition, the laboratory test design was established on 15 December 2024 at the University of Newcastle, Callaghan campus. The planar dimension of the designated laboratory room is 5.265 m × 2.89 m. As discussed earlier, four terrestrial laser scanners were employed under identical data acquisition conditions, and a Macbeth color chart containing 24 colors was used as the color reference (Figure 7).

Given the Macbeth color chart, Table 2 compares the normalized standard intensity values obtained from the standard RGB values of each color using the perceptual intensity equation (Appendix A).

The first scanner setup was established at the maximum room width, with the scanner oriented orthogonally to the center of each color patch. This is considered the ideal case for minimizing both the incidence angle and the circular spot size of the laser pulse. For the second setup, the inclined condition of scanning was proposed with the large incidence angle (greater than 60°) to the centre of the Macbeth color chart at the maximum room length. This is aimed at maximizing the incidence angle and the elliptical effect of the spot size of the laser pulse (i.e., an incidence angle larger than 60° significantly affects the precision of the 3D point cloud, resulting in approximately 20% more noise [4]). The other important consideration of the data collection operation is that the testing experiment was executed in a dark room with no interference from any external light. Every window, door, and seam of the room were completely sealed. Furthermore, the illuminated areas on the scanners, including their screen, were covered when data capturing to avoid any external illumination. This prevents any unexpected interference on the emitted and reflected laser pulses. Thereafter, during scanning, the highest default resolution within each scanner was selected (Table 3).

All acquired scans were finalized under “Scans Only” conditions, meaning that scans were captured with no attached images to potentially colorize 3D point clouds. It specifies that a signal pulse is accompanied by its own intensity strength from a single point measurement. Since data collection was achieved in a very short range and in an isolated room, the effect of other optical occurrences, such as atmospheric effects along the laser path, is quite negligible [6].

However, achieving standard intensity values under controlled laboratory conditions is not straightforward, as results are influenced by variations in scanner setups, scanning configurations, ambient lighting, target properties, etc. (Appendix A). It is important to note that the RGB values listed in Table 2 represent the true color values of the Macbeth color chart, whereas under “Scan Only” conditions, the strength of the laser response does not exactly correspond to the true RGB values (an empirical comparison to support the fact will be elaborated in Section 6.1). Thus, to determine the appropriate reference for each acquired dataset, the residuals for each color target under ideal conditions of scanning (orthogonal condition) were compared against the normalized intensity values. The smallest residuals consistently corresponded to the neutral color patches. Accordingly, neutral colors were selected as ground-truth reference for the datasets, as they are generally less affected by color variation and ambient lighting. Therefore, those colors yield the most stable results under experimental conditions. For example, in the case of Leica ScanStation C10, Neutral 5 demonstrated the least variation compared to all other colors.

## 6. Results

### 6.1. Pre-Processing Stages

#### 6.1.1. Radiometric Comparison: Intensity vs. RGB Across Scanners

The datasets under identical experimental instructions, as explained, were captured by four scanners. At the first stage, the individual color patches were extracted using corresponding software: Leica Register 360 (Leica Geosystems, Heerbrugg, Switzerland) (https://leica-geosystems.com/products/laser-scanners/software/leica-cyclone/leica-cyclone-register-360) (accessed on 15 September 2025) for Leica devices and Trimble Business Centre (TBC) (Trimble, Westminster, CO, USA) (https://geospatial.trimble.com/de/products/software/trimble-business-center) (accessed on 15 September 2025) for the Trimble device. To reduce the influence of the outliers imposed during manual selection, intensity values within three standard deviations (±3σ) from the average intensity of each color patch were filtered out. This filtering corresponds to a 99.7% confidence level to ensure that the dataset represents the true intensity values for each patch.

Under the “Scan Only” mode used in this study, analysis is solely based on the laser signal pulse. Thus, the specified outputs as the RGB values for a single point do not essentially represent true color values. Instead, they are the result of pseudo-color mapping, where the recorded laser intensity is artificially mapped into the red R, green G, and blue B channels for visualization purposes. Consequently, these RGB values are alternative representations of intensity, rather than physically meaningful color measures. Therefore, they cannot be considered reliable indicators of graphical colorization.

Table 4 compares the standard deviations of the averaged intensity values—from both the measured intensity values and those converted from RGB values—against standard intensity values for each color patch (as shown in Table 2) under both scanning conditions. The conversion equation from RGB values to the normalized standard intensity is provided in Appendix A.

The variations in intensities between the scanners are primarily associated with the scanners’ inherent operating systems, the laser wavelength, physical signal characteristics, transmitted pulse power, and noise levels—all of which are relevant to the radiometric behavior of surface reflection. The Leica ScanStation C10 has shorter wavelength (532 nm), lower power for the transmitted laser (3R corresponds to 8 mW), and smaller beam divergence (0.09 mrad). This scanner is very sensitive to color intensity variations in comparison to the remaining scanners functioning with the longer wavelength (infrared domain) transmitting higher power for the laser (10 mW).

**Figure 8:**
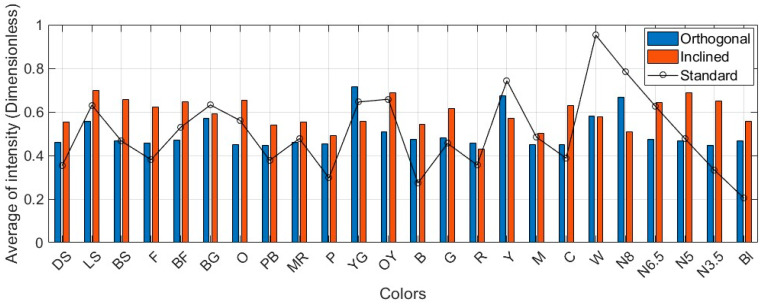
Average intensity values (dimensionless) for each color on the Macbeth color chart against the standard intensity values (dataset: Leica ScanStation C10). Plots for the other scanners are provided in Appendix B.

**Figure A1:**
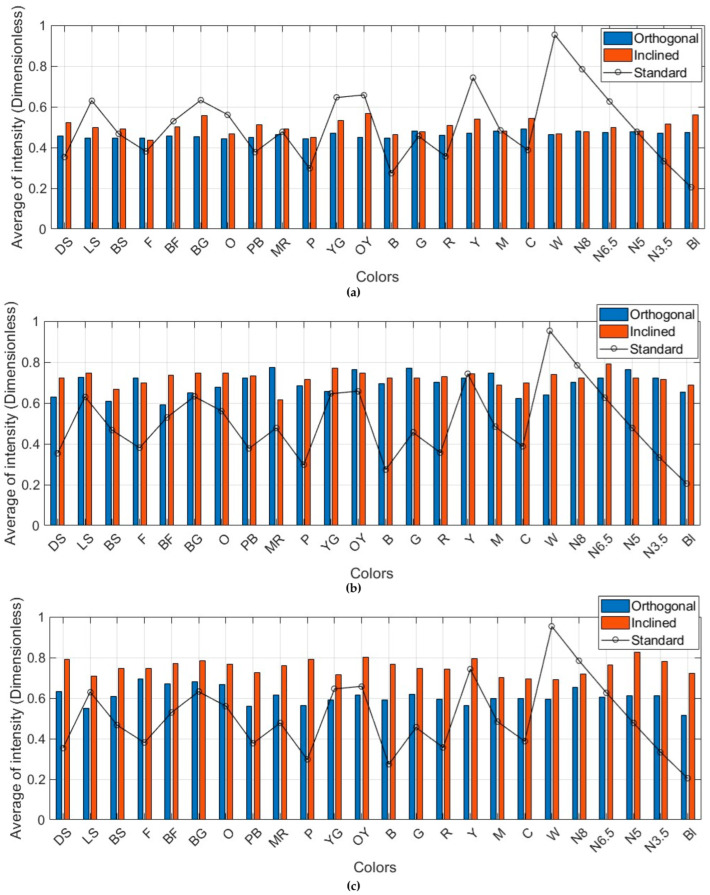
Average intensity values (dimensionless) for each color on the Macbeth color chart against the normalized standard values (dataset: (**a**) Leica ScanStation P50, (**b**) Leica RTC360, and (**c**) Trimble X9).

Results originated from the orthogonal scanning condition (presumably the ideal condition), as demonstrated in Figure 8 and Figure A1, vary with respect to the normalized standard intensities. As mentioned earlier, the standard intensities presented in Table 2 might not be optimally replicable even under the ideal scanning condition. Those are derived from true color values and are not identical to these converted from the pseudo-RGB measures. Moreover, the insignificant changes in range and incidence angle at the single-point level pose an additional challenge for consistent intensity modeling. To overcome this difficulty for quantitative precision assessment, within each dataset, one neutral color is selected as the reference color patch. For instance, Neutral 6.5 for Leica ScanStation C10 and Trimble X9 (Figure 8 and Figure A1c, respectively), Neutral 5 for Leica ScanStation P50 (Figure A1a), and Neutral 8 for Leica RTC360 (Figure A1b) were opted (i.e., Neutral colors are approximately invariant against variations in spatial and radiometric characteristics and record the minimal residuals with respect to their standard intensities).

In summary, the intensity value—as the fourth dimension attached to conventional 3D spatial point coordinates—is a numerical uncalibrated attribute for each point. Given the signal strength capacity, RGB values are the alternate representation of the intensity. In addition, the combined impact of the visible wavelength with smaller beam divergence in the Leica Scan Station C10 results in a larger diameter for the receiver to effectively adjust the level of illumination area from a reflected signal, as demonstrated in Equation (10). In contrast, the other scanners deliver not only less precise color intensities (Table 4) but also very consistent intensities (larger area of illumination) (Section 6.1.2).

#### 6.1.2. Reflectivity and Geometric Effects

In this section, the impact of three major factors, such as surface reflection and the individual geometric characteristics of the point, on the resulting intensity values is evaluated prior to implementing the proposed two-step method. The analyses here highlight that there is a general consistency in intensity among different color patches, with similar reflectivity patterns, whereas there is an inconsistency within the same patch due to minor variations in range and incidence angle among individual point measurements.

**Figure 9:**
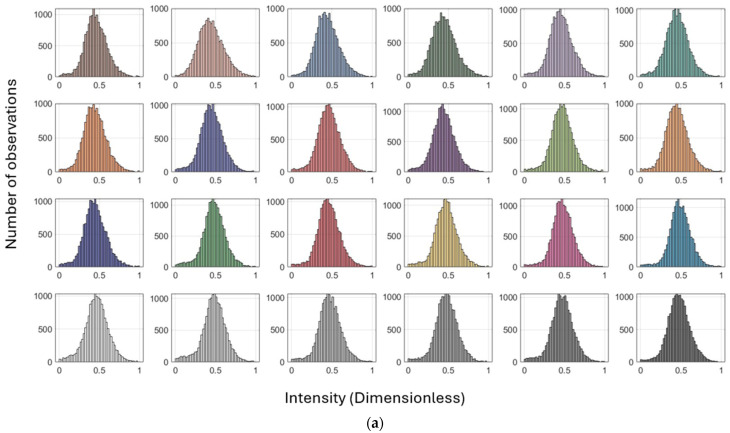

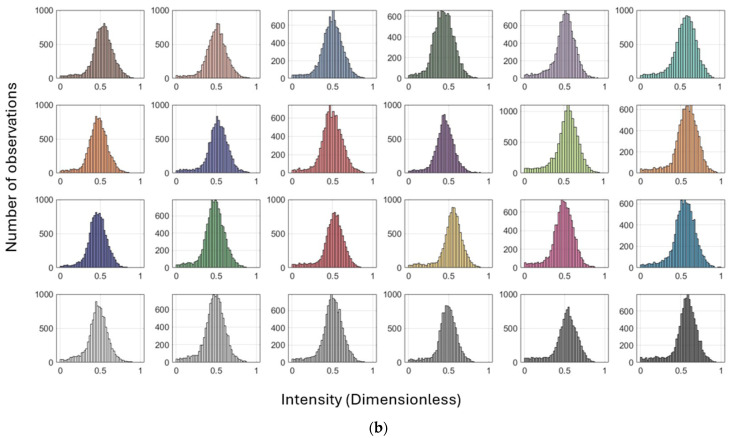
Histograms of intensity values versus the number of observations for each color on the Macbeth color chart (dataset: Leica ScanStation P50; scanning condition: (**a**) orthogonal and (**b**) inclined; and reference color patch: Neutral 5). Plots for the other scanners are provided in Appendix B.

**Figure A2:**
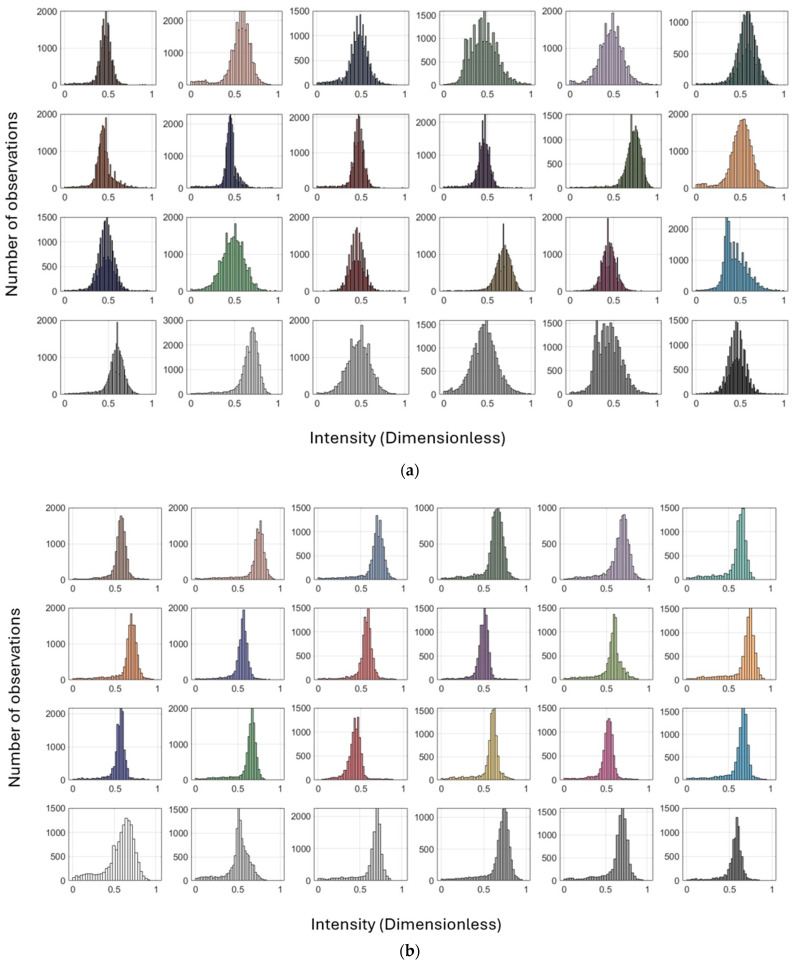
Histograms of intensities versus the number of observations for each color on the Macbeth color chart (dataset: Leica ScanStation C10; scanning condition: (**a**) orthogonal and (**b**) inclined; and reference color patch: Neutral 6.5).

**Figure A3:**
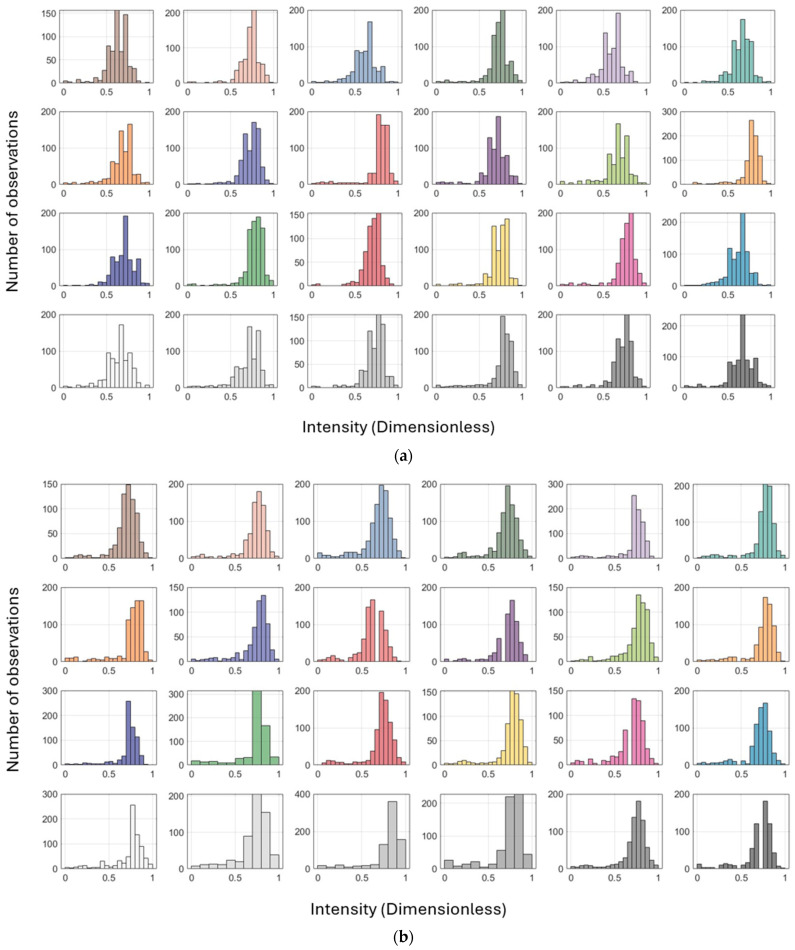
Histograms of intensities versus the number of observations for each color on the Macbeth color chart (dataset: Leica RTC360; scanning condition: (**a**) orthogonal and (**b**) inclined; and reference color patch: Neutral 8).

**Figure A4:**
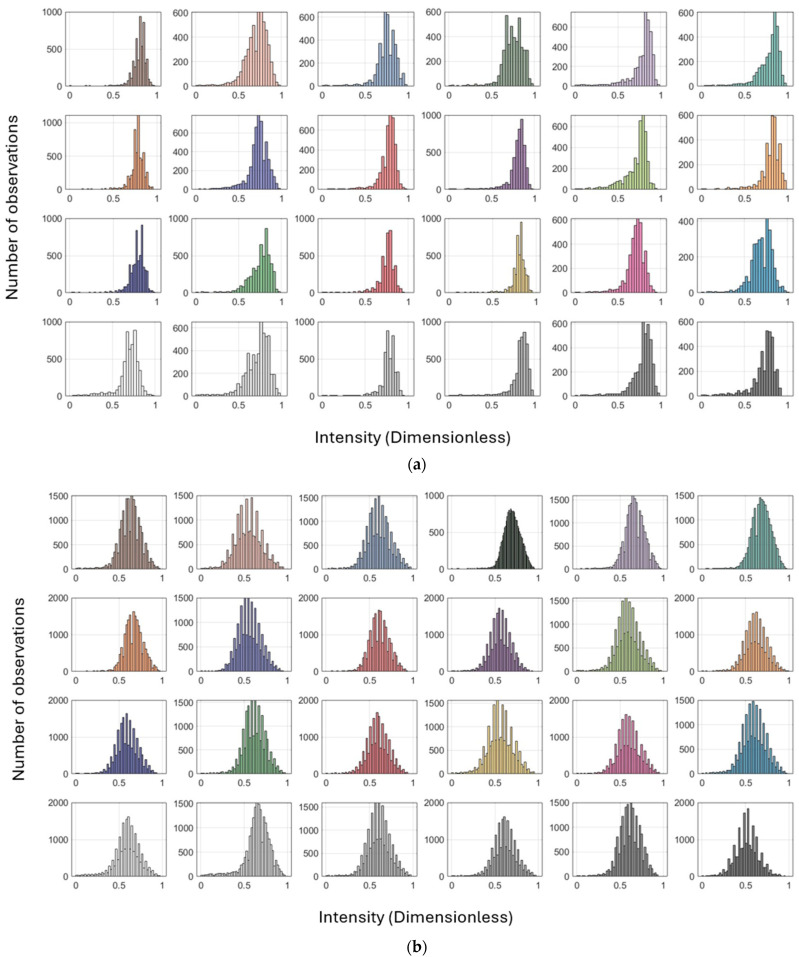
Histograms of intensities versus the number of observations for each color on the Macbeth color chart (dataset: Trimble X9; scanning condition: (**a**) orthogonal and (**b**) inclined; and reference color patch: Neutral 6.5).

Figure 9 and Table 5 illustrate the distribution of intensity values with respect to the number of point observations for each color patch on the Macbeth color chart for the Leica ScanStation P50 and the other scanners, respectively. These findings indicate that this consistency is the result of surface reflection determination, irrespective of the target quality (i.e., color). The same conditions occur for the other scanners (Figure A2, Figure A3 and Figure A4 in Appendix B).

The distribution shown in Figure 9 under the orthogonal scanning condition exhibits a symmetric pattern, indicating that signal strength is least affected by variations in range and incidence angle when measurements are taken orthogonally (i.e., normal Gaussian distribution). In contrast, the distribution under the inclined condition (at incidence angles greater than 60°) shows a non-symmetric pattern, where a larger number of points with weaker intensity produce a longer left tail, while fewer observations with stronger signal strength form a shorter right tail (i.e., skewed Gaussian distribution). Consequently, the impact of geometric effects, range, and incidence angle appears unpredictable. A similar behavior occurred for the other scanners (Figure A2, Figure A3 and Figure A4 in Appendix B).

The variation in point-to-point intensity behavior within the same color patch in Figure 9 is largely attributable to differences in geometric range and incidence angle, despite identical reflectivity patterns. This discrepancy is evident not only for the Leica ScanStation C10 but also for all other colors scanned by different scanners (Table 6).

In summary, the arguments above emphasize the complexity of radiometric calibration of TLS—the consistency of intensities across different color qualities and the inconsistency of intensities among individual points within the same color quality. To address these challenges, the behavior of individual points must be considered to enhance accuracy for a clearer distinction between color-dependent reflectivity (intensity) (Section 6.2) and to reduce the sensitivity of the range and incidence angle on point reflectivity (precision), leading to consistent (standardized) intensity values (Section 6.3).

### 6.2. Pointwise Radiometric Calibration Using LiDAR Range Equation

The pre-processing analyses emphasized the importance of pointwise intensity modeling for 4D TLS radiometric calibration, as intensity values of individual points are influenced by variations in three factors—reflectivity, range, and incidence angle. This implies that reflectivity, with respect to the target texture (i.e., color dependency), must be integrated into the pointwise geometric calibration practice. To achieve this objective, the elaborated methodologies were developed using the LiDAR range equation through a refined determination of the LiDAR cross-section for the specific target quality (i.e., color). This ultimately guarantees the highest accuracy in reflectivity estimation and facilitates a clearer distinction of intensities among different color targets (Equations (7) and (8)).

The LiDAR range equation establishes the relative connection between received and transmitted energy power, incorporating two areal ratios to determine the intensity of the signal strength as follows:

(1) At the receiver (i.e., TLS) location, the area of the receiver relative to the effective average area illuminated by the reflection from the target $\pi r^{2}$
(2) At the target location, the area of the LiDAR cross-section relative to the illumination area (i.e., particular attention must be drawn to determining the non-physical area of cross-section).

**Figure 10:**
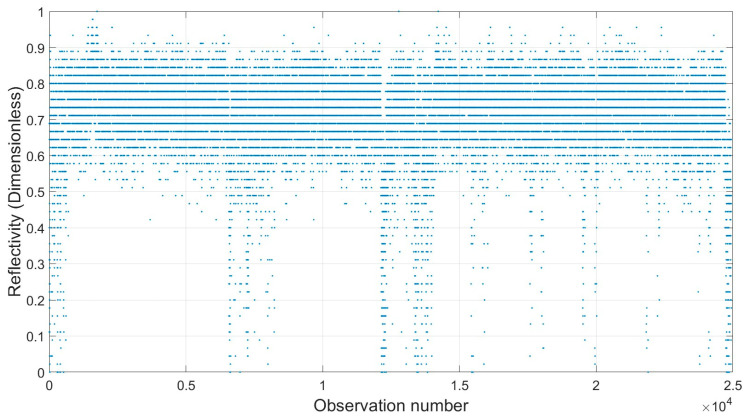
Relationship between individual observed point and reflectively (dimensionless) (dataset: Leica ScanStation P50; scanning condition: orthogonal; and color: yellow).

As an example, for a randomly selected color (yellow), the variation of reflectivity, as the major influential radiometric factor for individual point intensity observations under the orthogonal scanning condition for the Leica ScanStation P50, is shown (Figure 10).

A further analysis of the reflectivity patterns for the individual points across different colors on the Macbeth color chart under both scanning conditions demonstrated that the returned signal power is consistent across all colors, resulting in approximately identical reflectivity behavior. In other words, the intensities obtained from the scanners are insensitive and invariant to the color properties of the target. This uniformity can be attributed to surfaces exhibiting predominantly diffuse reflectance (100% diffuse reflectance ($\gamma_{d}\approx 1)$) (Equation (6)), producing similar responses for points with comparable textures. These support the adoption of a texture-dependent LiDAR cross-section technique to more effectively differentiate intensities across different surface targets (Equation (7)).

**Figure 11:**
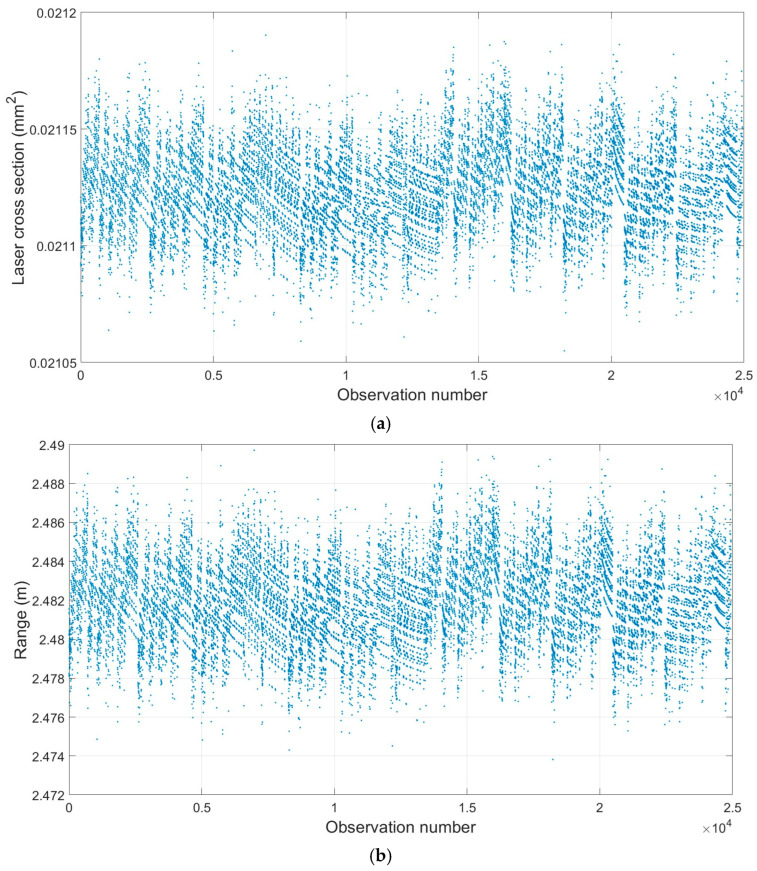

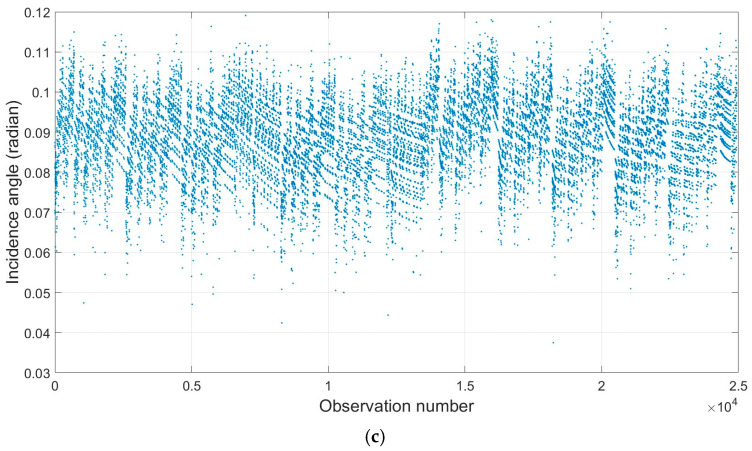
Relationship between individual observed point and (**a**) color-dependent LiDAR cross-section (${mm}^{2}$), (**b**) range (m), and (**c**) incidence angle (rad) (dataset: Leica ScanStation P50; scanning condition: orthogonal; and color: yellow). For further clarifications, the average color-dependent LiDAR cross-sections for each scanner are plotted under both scanning conditions (Figure A5 in Appendix C).

**Figure 12:**
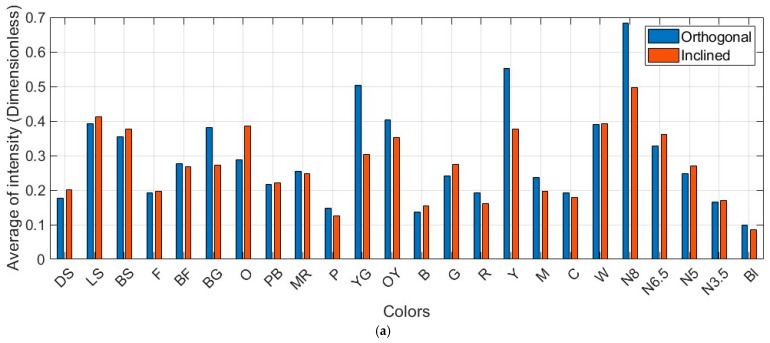

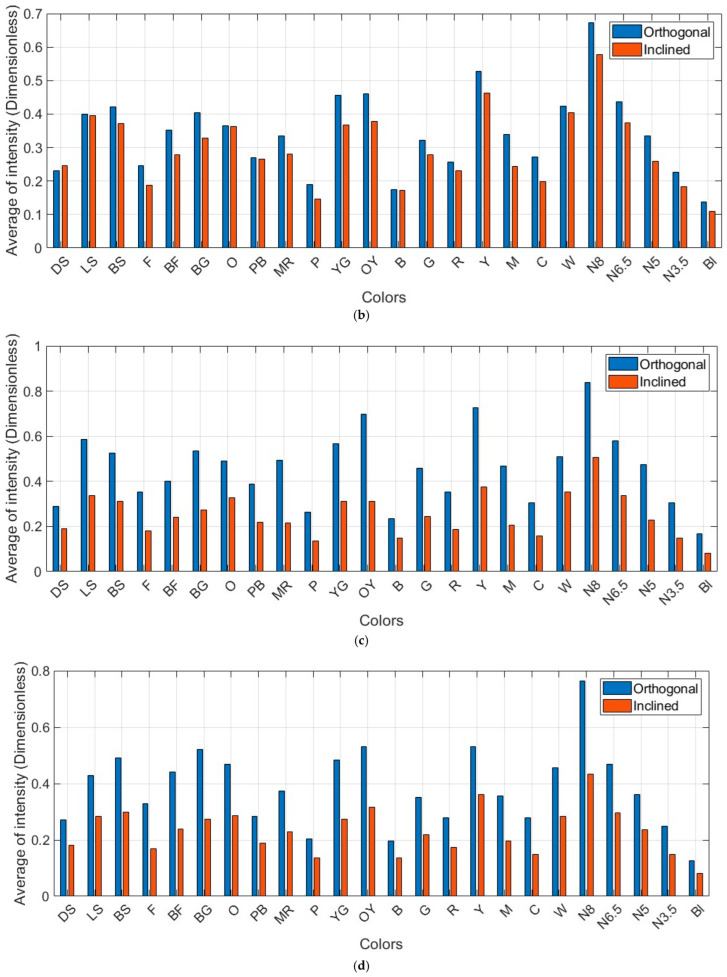
Average intensity values (dimensionless) for each color on the Macbeth color (dataset: (**a**) Leica ScanStation C10, (**b**) Leica ScanStation P50, (**c**) Leica RTC360, and (**d**) Trimble X9).

Considering the beam divergence and wavelength of the Leica ScanStation P50 (Table 1), combined with the standard intensity values of specific colors (Table 2), the color-dependent LiDAR cross-section area was determined exclusively for the points within the yellow patch (Figure 11). Given the comparison between the single-point LiDAR cross-section (Figure 11a) and the two other geometric components of the points (Figure 11b,c), the pointwise cross-section is more influenced by the range and incidence angle after the implementation of the approach. Importantly, the approach enables a better reproduction of color variations (Figure 12).

The uncertainties in each dataset before and after applying the implemented technique are listed (Table 7). The results deliver an acceptable level of improvement between 31% and 49% across different terrestrial laser scanners (Equation (13)). Interestingly, the results confirm the consistency of the proposed algorithm for the scanners operating at a shorter wavelength (Leica ScanStation C10) and at longer wavelengths (the remaining scanners). They demonstrate approximately identical accuracy afterward (0.09 and 0.10 for the orthogonal and inclined conditions, respectively). This outcome acknowledges the fact that the effect of geometric characteristics at the single-point level between the two scanning conditions has been addressed through the careful determination of the LiDAR range equation.

Here, to detach the color intensities in terms of the radiometric resolution in the TLS error model, the LiDAR range equation was proposed here to guarantee the required level of precision in reflectivity through the careful determination of the LiDAR cross-section. Thereafter, pointwise intensity modeling plays a critical role in achieving accurate intensity values for any sample target—not only for color properties, but also for other external formations and internal structures related to the targets. The following section elaborates on the standard radiometric practices through the weightings on the spatial range and incidence angle resolution of a single point observation. Clearly, the optimized values aim to attach real-world radiometric attributes to every single pulse as the fourth dimension of the 3D spatial point cloud.

### 6.3. Pointwise Radiometric Calibration Using a Neural Network

The radiometric calibration practice based on the amended LiDAR range equation—through effective application of the color-dependent LiDAR cross-section—shows a substantial accuracy improvement for pointwise intensity modeling—between 31% and 49% compared with the standard intensities of each color across four devices. These results assist in distinguishing intensity values according to the target characteristics (e.g., color) to better reflect individual color reprojection. However, more accurate intensity values do not necessarily generate consistent (precise) radiometric values within identical color quality. Additionally, the identical posteriori accuracy presented in Table 7 verifies the color-dependent LiDAR range equation partially elevates the precision by reducing the dependency between intensity and target–scanner geometry.

**Figure 13:**
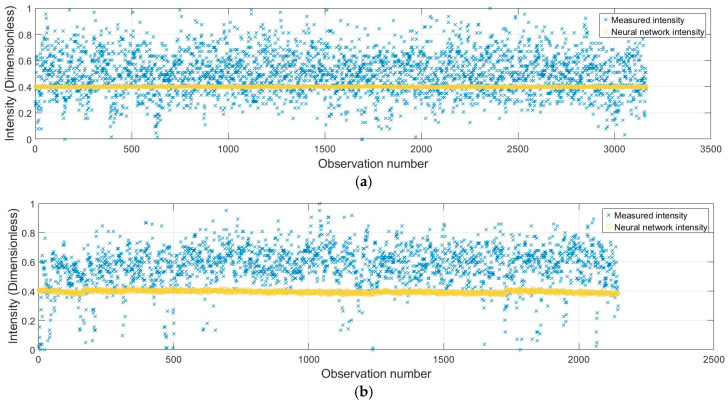
Pointwise intensity modeling using a data-driven neural network (dataset: Lecia ScanStation P50; color: yellow; and scanning condition: (**a**) orthogonal and (**b**) inclined).

For further validation, the developed data-driven neural network, implemented in four steps as described in the Methods section, was implemented on each acquired dataset, and precision evaluation was performed for each color relative to the neutral color patches selected as the reference patches. As an example, a comparison is illustrated between the point-to-point measured intensities and those derived from neural network prediction methods on a randomly selected color patch (yellow) from the Leica ScanStation P50 under both orthogonal and inclined conditions (Figure 13).

The findings reveal that the modifications in the reflectivity pattern of the surface according to the intrinsic color target and further correction (or validation) of the resulting values by neural network mitigate the impacts of the single point geometric range and incidence angle lying in the same color patch. The framework ultimately leads to precise (standard) intensity values for the single-color patch, incorporating all geometric and radiometric characteristics of the points. Secondly, identical intensities under both conditions of scanning demonstrate enhanced consistency for the selected color regardless of scanning conditions and target geometry.

**Figure 14:**
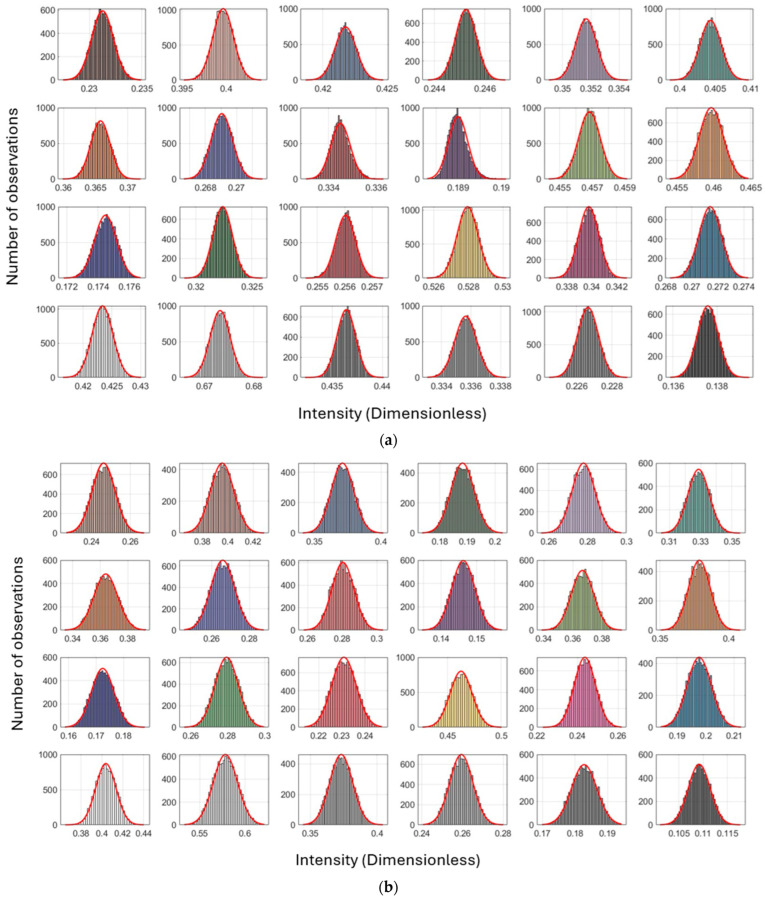
Histograms of intensities versus the number of observations for each color on the Macbeth color target (dataset: Leica ScanStation P50; scanning condition: (**a**) orthogonal and (**b**) inclined; and reference color patch: Neutral 5). Plots for the other scanners are provided in Appendix D.

Subsequently, the histograms for all colors are depicted in Figure 14. Compared to Figure 9, the results exhibit narrower standard deviations (Table 8). The pointwise calibration approaches a normal Gaussian distribution for both scanning conditions, rather than the previously skewed distribution in inclined scanning conditions, which is here inadequately influenced by the weaker response at a steep inclined angle. Table 8 summarizes the precision evaluation using Equation (13) for the intensities obtained through data-driven radiometric calibration. The improvements in precision are approximately 97% or higher in comparison with the measured intensities.

In summary, calibration strategies for the fourth dimension of the TLS (radiometric attribution) exhibit improvements of at least 31% in the accuracy of reflectivity estimation—before and after applying the LiDAR range equation—against the standard intensities of each color, and at least 97% in precision—before and after validation process using the data-driven neural network—against the neutral reference plane with the same dataset. These benefits are achieved by first addressing color-dependent reflectivity and subsequently minimizing the sensitivity of intensity values to variations in spatial properties. This approach standardizes intensity values for points sharing the identical radiometric characteristics but differing in spatial attributes. It further underlines the following conceptual calibration advancement in TLS error analysis: integrating 3D point coordinates of TLS can be complemented with the 1D intensity coordinate which provides a pathway towards fully standardized 4D point cloud calibration. This improvement carries future implications for TLS applications where radiometric consistency is essential, such as characterizing the internal properties (e.g., material and roughness) and external topographies (e.g., edges and tilts) of targets.

## 7. Discussions on Reflectivity (Intensity)

**Figure 15:**
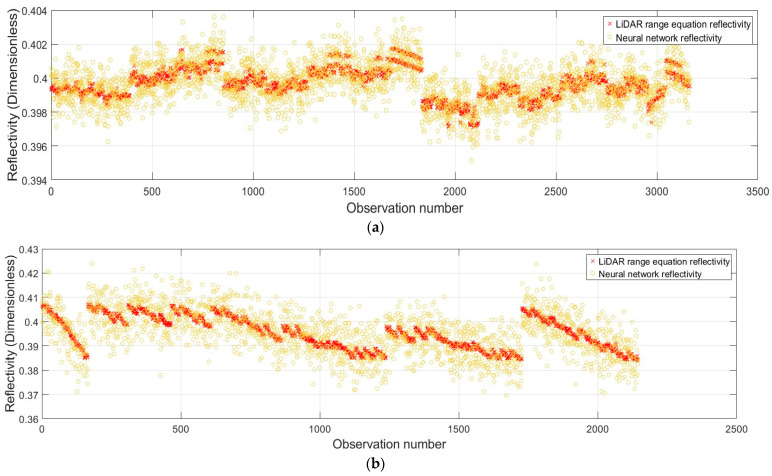
Pointwise reflectivity modeling (dataset: Lecia ScanStation P50; color: yellow; and scanning condition: inclined (**a**) orthogonal, and (**b**) inclined).

In this section, the comparison of the two proposed methodologies—color-dependent LiDAR range equation and data-driven neural network—is discussed in terms of validating the pointwise intensity results. As discussed, the results of the color-focused LiDAR range equation partially enhance the precision in Table 7. This summarizes that the reflectivity pattern is the dominant factor. The similarity in the reflectivity patterns, as shown in Figure 15, represents that the data-driven approach can reliably reproduce the physically characterized parameterization of the LiDAR range equation within the reasonable standard variations (i.e., here, it refers to as the color reproduction from the signal pulse). Accordingly, it is noticeable that some points recorded a very irregular pattern of reflection, even after applying a 99.7% confidence level for outlier detection. This is attributed to the irregular impact of range and incidence angle on single-point reflectivity, as expected. Due to this, the data-driven method is applied to create homogeneity by imposing spatial weightings. Secondly, those might be the effects of other optical elements, such as scattering, refraction, etc. These might be future-involving parameters in the 4D calibration study. In general, comparable trends occurred for the remaining colors across different scanner systems and scanning conditions.

**Figure 16:**
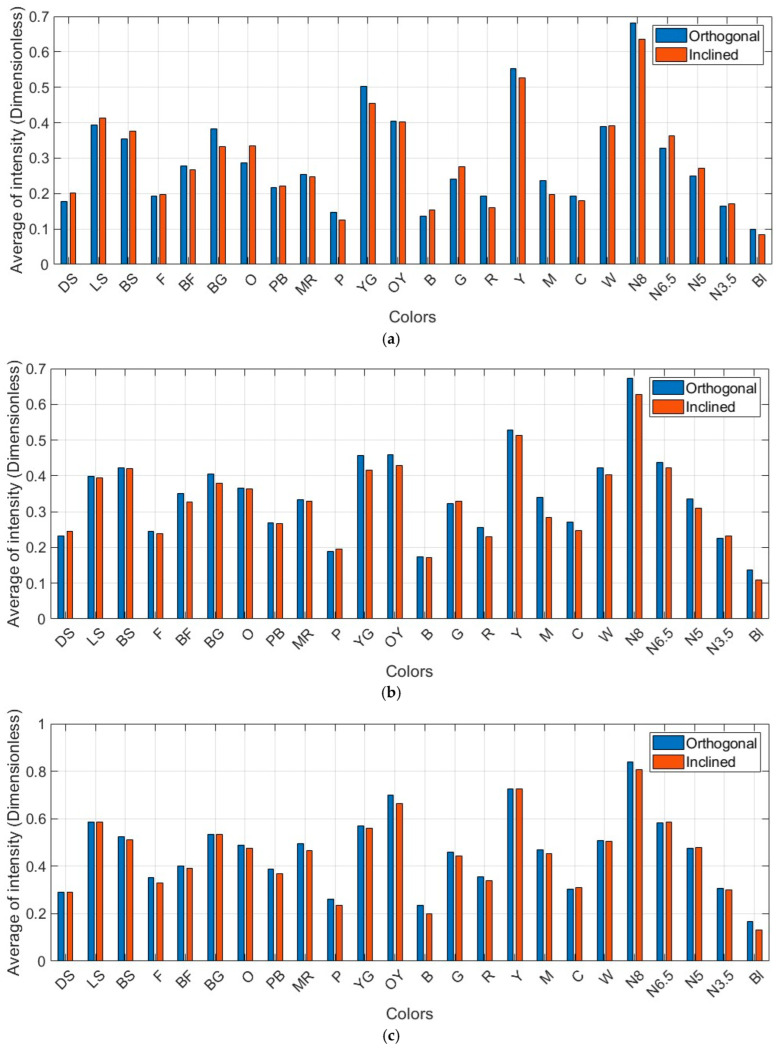

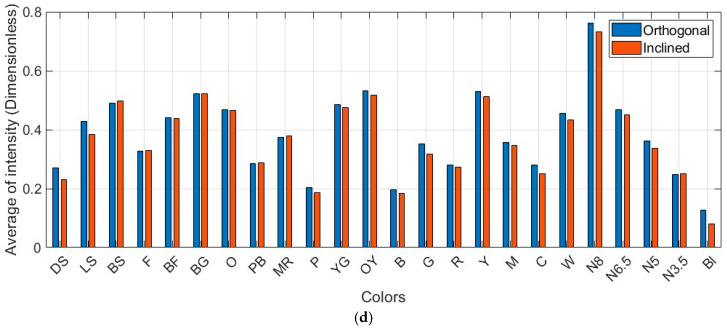
Average intensity values (dimensionless) for each color on the Macbeth color target (dataset: (**a**) Leica ScanStation C10, (**b**) Leica ScanStation P50, (**c**) Leica RTC360, and (**d**) Trimble X9).

The proximity of the average intensities under two varying scanning conditions outlines that the spatial weights applied to the geometric range and incidence angle of an individual point, incorporated with the corresponding reflectivity of the individual points through surface-dependency reflection, bring positive outcomes (Figure 16). Particularly, these findings enable us to reduce the variabilities that were recorded as significantly high at larger incidence angles (i.e., the equal consistent intensities for each color regardless of the geometric target/scanning conditions).

Overall, the results from the proposed calibration framework address the following two key scenarios: (1) improved real-world reflectivity characterization, and (2) more precise (consistent) intensity values across different target texture qualities. For the first aspect, since reflectivity is often the primary indicator for identifying the internal characteristics of a target, a thorough understanding of the laser cross-section for any given target can significantly enhance the accurate determination of the received power amount. For example, in applications such as soil moisture detection, vegetation health assessment, surface material classification, and monitoring of painted or coated infrastructures (e.g., bridges, buildings, or road markings), texture-dependent reflectivity according to the signal pulse plays a critical role in distinguishing between radiometric variations. For the second aspect, the framework supports calibrated intensity as a reliable fourth dimension of the point cloud (i.e., the robust foundation for standardized 4D point cloud analysis across various LiDAR devices with multiple measurement configurations obtained from different platforms).

## 8. Conclusions and Future Investigations

This study aimed to investigate accurate radiometric values for 3D point clouds. The fourth dimension of the point cloud is assumed to be the reliable representation of signal intensity. Multiple factors, such as range, incidence angle, and reflectivity, contribute to the signal intensity. Note that not all points belonging to the same target quality reflect the signal uniformly. Under the proposed methodology, the innovative pointwise radiometric calibration addresses the spatial and radiometric conditions of individual points in order to enhance the accuracy and precision of intensity values for the points that often have the same target characteristics, accounting for the inherent physical properties of the laser and target geometry for better color reproduction of the signal pulse.

As already expressed, previous studies focused on applying the mean intensity values for targets with the same texture (i.e., using fitted planes through the laser radar range equation), which totally ignore the significance of LiDAR cross-section determination and the spatial conditions of single point measurements. The current work, for the first time, concentrates on the individual point observation to obtain more accurate and precise intensities using the LiDAR range equation through the appropriate resolution of the color-dependent LiDAR cross-section. As the initial step, the reflectivity estimation is pursued to clarify standard and accurate reflectivity respective to color variations on the Macbeth color chart. Furthermore, by relying on developed neural network algorithms, the precision of each optimized value is verified, providing meaningful radiometric values attached to each color, taking into account the single pulse geometric conditions.

For the data collection steps, four terrestrial laser scanners—Leica ScanStation P50, Leica ScanStation C10, Leica RTC360 (Leica Geosystems, Heerbrugg, Switzerland), Trimble X9 (Trimble, Westminster, Colorado, USA)—were used. The highest default resolution of each scanner under “Scan Only” was chosen during scanning. In addition, the internal condition of the calibration room was isolated from any external illumination interference, and observations were acquired under similar procedures at two fixed locations as follows: orthogonal (presumed as the ideal condition) and inclined (incidence angle larger than 60°), scanning conditions with respect to the Macbeth color chart. Reasonable improvements were observed in the accuracy and precision of the intensity values for each color patch as follows: uncertainty improvements between 31% and 49% against the standard intensities of each color and precision improvements of 97% against the neutral reference plane with the same dataset across different scanner devices and scanning conditions. The implemented techniques introduce a novel standardization of intensity values for points sharing the equivalent color textures obtained from a changeable geometric range and incidence angle.

For future works, the entire proposed algorithm has the potential to be applied to a wide range of target geometries (e.g., internal object- and external surface-related features). This predominantly leads to visible distinctions associated with identical target geometries. To advance this in a more sophisticated manner, the auxiliary theoretical parameterization of the texture-dependent LiDAR cross-section is strongly recommended, particularly with reference to the proportion of incident energy returned by an ideal Lambertian surface. The Lambertian surface is assumed to theoretically simplify research activities; however, this must be complemented by the other optical phenomena which might be reflected as the noise shown in Figure 15.

## Figures and Tables

**Table 1:** Technical radiometric specifications of terrestrial laser scanners for this experiment.

Specifications	TLSs			
	Leica ScanStation P50 ^1^	Leica ScanStation C10 ^2^	Leica RTC360 ^3^	Trimble X9 ^4^
Laser class	1	3R	1	1
Wavelength	From 685 nm to 1550 nm	532 nm	1550 nm	From 1530 nm to 1570 nm
Initial beam diameter	≤3.5 mm (FWHM)	−	6 mm	−
Spot size *	−	From 0–50 m: 4.5 mm (FWHH-based); 7 mm (Gaussian-based)	−	7.95 mm at 10 m
Beam divergence	<0.23 mrad (FWHM, full angle)	0.09 mrad	0.5 mrad (full angle)	0.8 mrad

* The difference between spot size and initial beam diameter is that the spot size is the size of the laser pulse at the target location, while the initial beam diameter is introduced as the diameter of the beam at the transmitter location. The spot size can be converted to any given beam divergence ϑ in radians at any assumed range $\vartheta \approx \frac{spot\;size}{r}$. ^1^ https://leica-geosystems.com/products/laser-scanners/scanners/leica-scanstation-p50 (accessed on 15 September 2025). ^2^ https://leica-geosystems.com/-/media/files/leicageosystems/products/brochures/leica_scanstation_c5_bro.ashx?la=en (accessed on 15 September 2025). ^3^ https://leica-geosystems.com/products/laser-scanners/scanners/leica-rtc360 (accessed on 15 September 2025). ^4^ https://geospatial.trimble.com/de/links?dcs=Collection-133328 (accessed on 15 September 2025).

**Table 2:** Standard intensity values for each color on the Macbeth color chart (dimensionless).

Standard Intensity Values					
Dark skin (DS)	Light skin (LS)	Blue sky (BS)	Foliage (F)	Blue flower (BF)	Bluish green (BG)
0.35	0.63	0.65	0.38	0.53	0.62
Orange (O)	Purplish blue (PB)	Moderate red (MR)	Purple (P)	Yellow green (YG)	Orange yellow (OY)
0.57	0.41	0.49	0.29	0.66	0.70
Blue (B)	Green (G)	Red (R)	Yellow (Y)	Magenta (M)	Cyan (C)
0.27	0.46	0.38	0.76	0.48	0.38
White (W)	Neutral 8 (N8)	Neutral 6.5 (N6.5)	Neutral 5 (N5)	Neutral 3.5 (N3.5)	Black (Bl)
0.95	0.79	0.63	0.48	0.33	0.20

**Table 3:** The highest default resolution employed for the data experiments.

TLSs			
Leica ScanStation P50	Leica ScanStation C10	Leica RTC360	Trimble X9
0.8 mm at 10 m	0.002 m at 10 m	3 mm at 10 m	3 mm at 10 m

**Table 4:** Comparison of standard deviations (±1σ) between the observed radiometric values.

Scanning Conditions	Standard Deviation	TLSs			
		Leica ScanStation P50	Leica ScanStation C10	Leica RTC360	Trimble X9
Orthogonal	Measured intensity	0.179	0.134	0.168	0.176
	Computed intensity from RGB	0.178	0.132	0.167	0.176
Inclined	Measured intensity	0.181	0.179	0.187	0.189
	Computed intensity from RGB	0.182	0.173	0.189	0.189

**Table 5:** Average intensity values with the corresponding number of points (NP) for each color target on the Macbeth color chart.

TLSs	Leica ScanStation P50				Leica ScanStation C10				Leica RTC360				Trimble X9			
SC ^1^/NP	O	NP	I	NP	O	NP	I	NP	O	NP	I	NP	O	NP	I	NP
DS	0.458	16,328	0.523	11,227	0.459	25,591	0.554	12,461	0.630	0.137	0.724	681	0.632	18,976	0.791	5425
LS	0.446	15,816	0.499	10,723	0.558	25,900	0.700	11,654	0.726	0.128	0.747	763	0.550	18,923	0.709	5262
BS	0.447	16,148	0.492	10,328	0.467	26,096	0.656	10,116	0.609	0.146	0.668	988	0.607	19,181	0.745	4042
F	0.447	16,285	0.437	10,419	0.457	24,021	0.624	9656	0.721	0.145	0.698	840	0.694	19,381	0.745	4457
BF	0.458	16,548	0.500	10,258	0.470	24,969	0.647	8895	0.591	0.132	0.735	814	0.669	19,298	0.772	4049
BG	0.452	16,422	0.558	9907	0.570	24,557	0.593	7943	0.651	0.128	0.748	796	0.682	19,083	0.785	3386
O	0.444	16,271	0.468	10,988	0.451	25,168	0.655	13,098	0.677	0.147	0.747	729	0.669	18,803	0.768	5880
PB	0.451	16,560	0.513	10,941	0.448	26,204	0.541	11,434	0.724	0.123	0.732	561	0.559	18,868	0.725	5128
MR	0.464	16,823	0.490	10,242	0.461	26,274	0.554	9754	0.773	0.164	0.616	755	0.615	19,363	0.761	3981
P	0.442	16,952	0.451	10,323	0.452	26,233	0.490	9753	0.684	0.152	0.714	628	0.563	19,362	0.791	4632
YG	0.471	16,750	0.534	10,454	0.716	25,253	0.559	9259	0.657	0.155	0.770	593	0.590	19,225	0.715	3992
OY	0.452	16,288	0.568	9899	0.510	25,230	0.690	7782	0.764	0.129	0.745	679	0.615	18,989	0.803	3222
B	0.446	16,440	0.463	10,927	0.475	24,375	0.543	12,840	0.695	0.126	0.722	699	0.592	18,791	0.767	5761
G	0.481	16,702	0.478	10,826	0.481	24,354	0.617	11,926	0.770	0.132	0.721	641	0.619	19,131	0.748	5275
R	0.462	16,605	0.508	10,635	0.457	25,515	0.430	9544	0.703	0.125	0.728	801	0.595	19,214	0.742	3996
Y	0.472	16,850	0.539	10,877	0.673	24,938	0.571	10,088	0.723	0.129	0.744	632	0.564	19,192	0.795	4704
M	0.479	16,596	0.481	10,361	0.450	24,659	0.503	9151	0.748	0.145	0.686	585	0.599	19,252	0.702	4008
C	0.491	16,557	0.542	9781	0.450	24,724	0.630	7927	0.623	0.131	0.697	705	0.597	18,979	0.696	3151
W	0.465	16,595	0.466	11,176	0.580	24,589	0.579	12,668	0.640	0.148	0.739	757	0.596	18,539	0.690	5571
N8	0.480	16,798	0.479	10,932	0.667	24,921	0.509	11,808	0.703	0.152	0.721	574	0.654	19,015	0.719	5255
N6.5	0.473	16,659	0.499	10,467	0.474	24,264	0.643	10,298	0.723	0.136	0.790	756	0.605	19,192	0.763	4155
N5	0.477	16,705	0.481	10,475	0.466	24,543	0.689	10,031	0.762	0.164	0.724	640	0.612	19,140	0.825	4731
N3.5	0.470	16,682	0.515	10,594	0.445	24,674	0.650	9130	0.722	0.138	0.715	742	0.612	19,049	0.780	3870
Bl	0.476	16,880	0.559	10,332	0.467	24,588	0.557	7969	0.655	0.152	0.688	620	0.516	18,833	0.723	3485

^1^ SC stands for scanning conditions, including two types, Orthogonal (O) and Inclined (I).

**Table 6:** Precision (±1σ) of intensity for each color target on the Macbeth color chart.

TLSs	Leica ScanStation P50		Leica ScanStation C10		Leica RTC360		Trimble X9	
Scanning Conditions	Orthogonal	Inclined	Orthogonal	Inclined	Orthogonal	Inclined	Orthogonal	Inclined
Dark skin (DS)	0.142	0.144	0.099	0.107	0.137	0.147	0.128	0.107
Light skin (LS)	0.158	0.137	0.143	0.161	0.128	0.172	0.151	0.132
Blue sky (BS)	0.147	0.141	0.127	0.154	0.146	0.168	0.139	0.129
Foliage (F)	0.155	0.133	0.152	0.138	0.145	0.159	0.109	0.128
Blue flower (BF)	0.149	0.140	0.141	0.146	0.132	0.177	0.119	0.165
Bluish green (BG)	0.144	0.156	0.121	0.150	0.128	0.169	0.119	0.143
Orange (O)	0.144	0.130	0.115	0.145	0.147	0.191	0.114	0.103
Purplish blue (PB)	0.144	0.143	0.108	0.108	0.123	0.172	0.134	0.130
Moderate red (MR)	0.144	0.139	0.103	0.113	0.164	0.161	0.120	0.117
Purple (P)	0.141	0.127	0.104	0.099	0.152	0.163	0.126	0.128
Yellow green (YG)	0.142	0.157	0.125	0.143	0.155	0.165	0.138	0.149
Orange yellow (OY)	0.145	0.152	0.135	0.166	0.129	0.172	0.135	0.129
Blue (B)	0.145	0.122	0.109	0.108	0.126	0.151	0.129	0.112
Green (G)	0.144	0.135	0.134	0.139	0.132	0.194	0.129	0.139
Red (R)	0.142	0.142	0.098	0.099	0.125	0.173	0.131	0.121
Yellow (Y)	0.143	0.143	0.116	0.135	0.129	0.170	0.146	0.115
Magenta (M)	0.136	0.140	0.096	0.114	0.145	0.179	0.138	0.120
Cyan (C)	0.137	0.148	0.137	0.146	0.131	0.170	0.141	0.132
White (W)	0.149	0.135	0.118	0.172	0.148	0.188	0.146	0.133
Neutral 8 (N8)	0.149	0.137	0.129	0.143	0.152	0.178	0.146	0.149
Neutral 6.5 (N6.5)	0.142	0.137	0.133	0.160	0.136	0.200	0.136	0.123
Neutral 5 (N5)	0.142	0.127	0.149	0.153	0.164	0.208	0.135	0.143
Neutral 3.5 (N3.5)	0.143	0.155	0.142	0.153	0.138	0.170	0.127	0.145
Black (Bl)	0.140	0.155	0.113	0.120	0.152	0.180	0.134	0.152

**Table 7:** Accuracy (±1σ) of intensity with respect to the standard intensities before and after the adoption of the LiDAR range equation.

Scanning Conditions	Accuracy		TLSs		
		Leica ScanStation P50	Leica ScanStation C10	Leica RTC360	Trimble X9
Orthogonal	Before	0.178	0.134	0.187	0.176
	After	0.093	0.093	0.096	0.095
	**Improvement**	**48%**	**31%**	**49%**	**46%**
Inclined	Before	0.182	0.179	0.167	0.189
	After	0.097	0.103	0.104	0.116
	**Improvement**	**47%**	**42%**	**38%**	**39%**

**Table 8:** Precision (±1σ) of intensities with respect to neutral colors in each dataset after data-driven neural network adoption.

TLSs	Leica ScanStation P50		Leica ScanStation C10		Leica RTC360		Trimble X9	
Scanning Conditions	Orthogonal	Inclined	Orthogonal	Inclined	Orthogonal	Inclined	Orthogonal	Inclined
Dark skin (DS)	0.001	0.006	0.001	0.005	0.001	0.003	0.001	0.002
Light skin (LS)	0.001	0.009	0.002	0.011	0.002	0.005	0.002	0.003
Blue sky (BS)	0.001	0.009	0.001	0.010	0.001	0.006	0.001	0.003
Foliage (F)	0.000	0.004	0.001	0.005	0.000	0.003	0.000	0.002
Blue flower (BF)	0.001	0.006	0.001	0.006	0.000	0.004	0.001	0.002
Bluish green (BG)	0.001	0.007	0.002	0.006	0.001	0.004	0.002	0.003
Orange (O)	0.001	0.009	0.001	0.010	0.002	0.005	0.002	0.003
Purplish blue (PB)	0.001	0.006	0.001	0.005	0.001	0.003	0.001	0.002
Moderate red (MR)	0.000	0.006	0.001	0.006	0.001	0.003	0.001	0.003
Purple (P)	0.000	0.003	0.000	0.003	0.000	0.002	0.000	0.001
Yellow green (YG)	0.001	0.008	0.001	0.007	0.001	0.005	0.001	0.003
Orange yellow (OY)	0.002	0.008	0.002	0.008	0.002	0.005	0.002	0.003
Blue (B)	0.001	0.004	0.001	0.004	0.001	0.002	0.001	0.001
Green (G)	0.001	0.006	0.001	0.007	0.002	0.004	0.001	0.002
Red (R)	0.000	0.005	0.001	0.004	0.001	0.003	0.001	0.002
Yellow (Y)	0.001	0.010	0.001	0.009	0.002	0.005	0.001	0.004
Magenta (M)	0.001	0.005	0.001	0.004	0.002	0.003	0.001	0.002
Cyan (C)	0.001	0.004	0.001	0.004	0.001	0.003	0.001	0.002
White (W)	0.002	0.010	0.002	0.010	0.003	0.005	0.002	0.003
Neutral 8 (N8)	0.002	0.013	0.003	0.012	0.004	0.009	0.003	0.004
Neutral 6.5 (N6.5)	0.001	0.009	0.001	0.009	0.002	0.005	0.001	0.003
Neutral 5 (N5)	0.001	0.006	0.001	0.006	0.002	0.004	0.001	0.002
Neutral 3.5 (N3.5)	0.001	0.004	0.001	0.004	0.001	0.002	0.001	0.002
Black (Bl)	0.000	0.002	0.000	0.002	0.001	0.001	0.000	0.001

**Figure A5:**
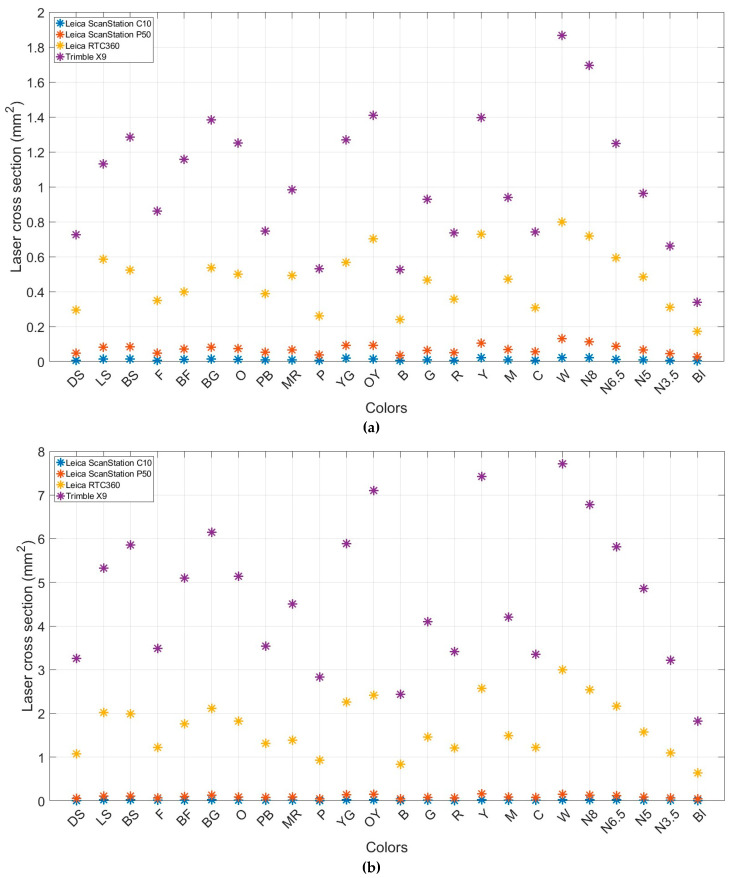
Average color-dependent LiDAR cross-section (${mm}^{2}$) (scanning condition: (**a**) orthogonal and (**b**) inclined).

**Figure A6:**
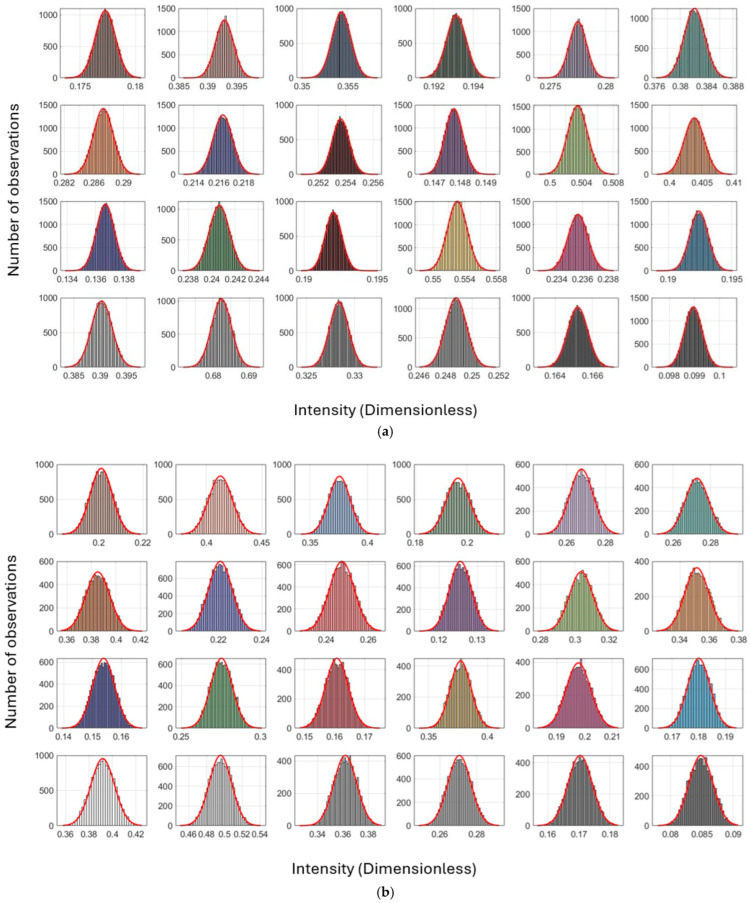
Histograms of intensities versus the number of observations for each color on the Macbeth color chart (dataset: Leica ScanStation C10; scanning condition: (**a**) orthogonal and (**b**) inclined; and reference color patch: Neutral 6.5).

**Figure A7:**
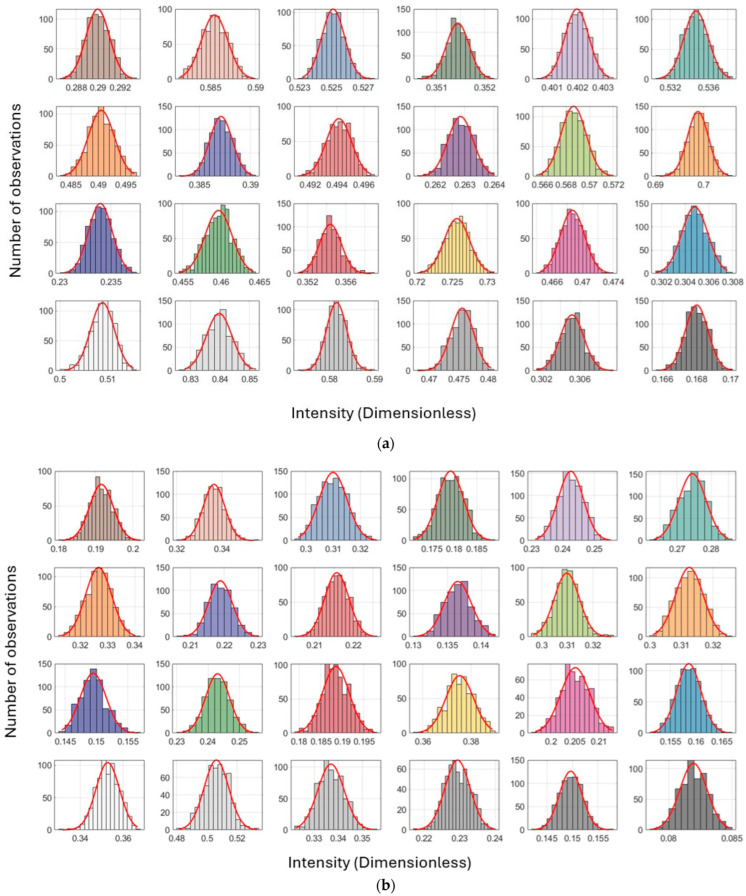
Histograms of intensities versus the number of observations for each color on the Macbeth color chart (dataset: Leica RTC360; scanning condition: (**a**) orthogonal and (**b**) inclined; and reference color patch: Neutral 8).

**Figure A8:**
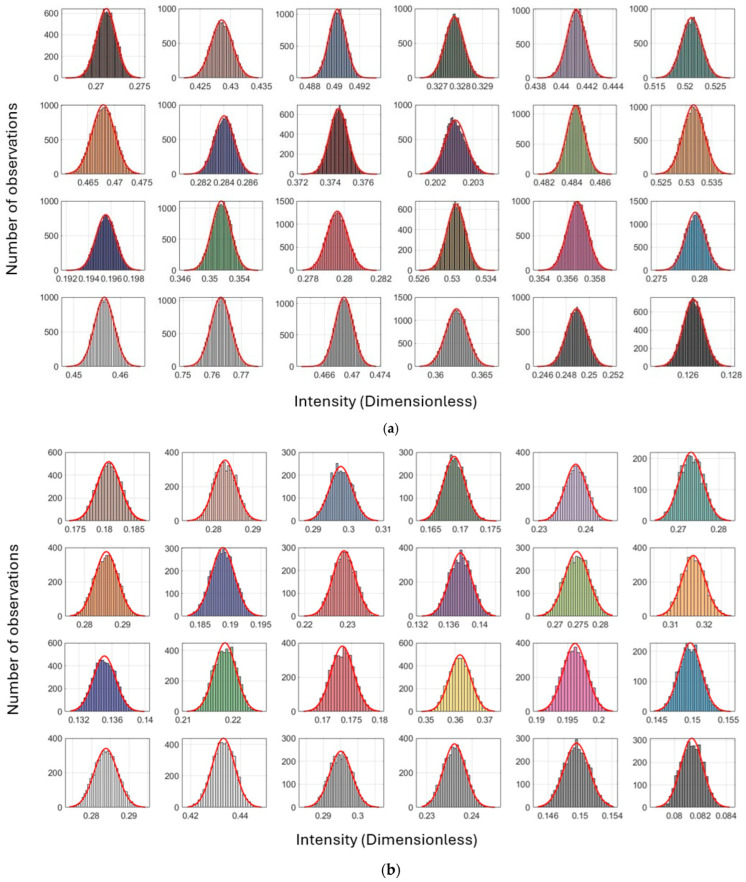
Histograms of intensities versus the number of observations for each color on the Macbeth color chart (dataset: Trimble X9; scanning condition: (**a**) orthogonal and (**b**) inclined; and reference color patch: Neutral 6.5).

## Data Availability

The original contributions presented in this study are included in the article. Further inquiries can be directed to the corresponding author(s).

## Appendix A. Perceptual Intensity Equation

The RGB values can be converted to perceptual intensity I using the following weighted normalized equation:

(A1) $$I=\frac{0.2989\;R+0.587\;G+0.114\;B}{255},$$
